# A novel derivative of betulinic acid, SYK023, suppresses lung cancer growth and malignancy

**DOI:** 10.18632/oncotarget.3701

**Published:** 2015-03-30

**Authors:** Tsung-I Hsu, Ying-Jung Chen, Chia-Yang Hung, Yi-Chang Wang, Sin-Jin Lin, Wu-Chou Su, Ming-Derg Lai, Sang-Yong Kim, Qiang Wang, Keduo Qian, Masuo Goto, Yu Zhao, Yoshiki Kashiwada, Kuo-Hsiung Lee, Wen-Chang Chang, Jan-Jong Hung

**Affiliations:** ^1^ Center for Infection Disease and Signal Research, College of Medicine, National Cheng Kung University, Tainan, Taiwan; ^2^ Institute of Bioinformatics and Biosignal Transduction, College of Bioscience and Biotechnology, National Cheng Kung University, Tainan, Taiwan; ^3^ Institute of Basic Medical Sciences, College of Medicine, National Cheng Kung University, Tainan, Taiwan; ^4^ Department of Internal Medicine, College of Medicine and Hospital, National Cheng Kung University, Tainan, Taiwan; ^5^ Department of Biochemistry and Molecular Biology, College of Medicine, National Cheng Kung University, Tainan, Taiwan; ^6^ Natural Products Research Laboratories, UNC Eshelman School of Pharmacy, University of North Carolina, Chapel Hill, NC, USA; ^7^ Laboratory of Pharmacognosy, Graduate School of Pharmaceutical Sciences, The University of Tokushima, Tokushima, Japan; ^8^ Chinese Medicine Research and Development Center, China Medical University and Hospital, Taichung, Taiwan; ^9^ Department of Pharmacology, College of Medicine, National Cheng Kung University, Tainan, Taiwan; ^10^ Graduate Institute of Medical Sciences, College of Medicine, Taipei Medical University, Taipei, Taiwan

**Keywords:** SYK023, ER stress, metastasis, synaptopodin

## Abstract

Herein, we evaluated the anti-cancer effect and molecular mechanisms of a novel betulinic acid (BA) derivative, SYK023, by using two mouse models of lung cancer driven by Kras^G12D^ or EGFR^L858R^. We found that SYK023 inhibits lung tumor proliferation, without side effects *in vivo* or cytotoxicity in primary lung cells *in vitro*. SYK023 triggered endoplasmic reticulum (ER) stress. Blockage of ER stress in SYK023-treated cells inhibited SYK023-induced apoptosis. In addition, we found that the expression of cell cycle-related genes, including cyclin A2, B1, D3, CDC25a, and CDC25b decreased but, while those of p15^INK4b^, p16^INK4a^, and p21^CIP1^ increased following SYK023 treatment. Finally, low doses of SYK023 significantly decreased lung cancer metastasis *in vitro* and *in vivo*. Expression of several genes related to cell migration, including synaptopodin, were downregulated by SYK023, thereby impairing F-actin polymerization and metastasis. Therefore, SYK023 may be a potentially therapeutic treatment for metastatic lung cancer.

## INTRODUCTION

Betulinic acid (BA), a natural pentacyclic triterpene, has a variety of beneficial biological activities, including anti-tumor, anti-viral, anti-bacterial, anti-malarial and anti-inflammatory activity [1, 2]. BA can suppress tumorigenesis in several types of cancer, including lung, colon, breast, pancreatic, and prostate cancers [3-7]. It has been shown to inhibit tumor growth by inducing mitochondria-dependent apoptosis [1]. However, BA also inhibits the expression of tumor-promoting proteins, including lamin B1, ErbB2, Stat3, HIF-1α and Sp1 [5, 6, 8]. Further, we have shown that BA inhibits lung tumor growth through the inhibition of Sp1-mediated cyclin A2 expression *in vitro* and *in vivo* [4]. Given the effectiveness of BA in inhibiting tumorigenesis, there is interest in developing novel derivatives to enhance its benefits, while minimizing side effects. Previous work has shown that a cyano derivative of BA potently inhibits colon and pancreatic cancer. [9]. *N*-Acylimidazole and C-3 carbamate derivatives also enhance the anti-proliferative effect of BA in HepG2 cells [10]. Moreover, 2,4-dinitrophenylhydrazone BA derivatives enhance cytotoxicity in melanoma, anaplastic thyroid tumors, and ovarian, lung, and breast cancer cell lines [11]. 3-*O*-Propargylated BA containing 1,2,3-triazoles displays a strong anti-tumor effect in leukemia cell lines [12]. In addition, ionic and glycosylated BA derivatives display increased solubility and improve the anti-tumor effect of BA [13, 14]. Our previous study also indicated that 20 mg/kg of BA can inhibit lung tumor growth in the Kras^G12D^-induce lung cancer mice. However, this dose is higher than other clinical or pre-clinical used drug. In addition, our preliminary results also show that highly frequency of lethality occurred after BA injection, suggesting that BA cause high cytotoxicity toward normal cell leadings side-effect. Based on these studies, to screen the BA derivative(s) with higher anti-tumor activity and low side-effect is crucial for lung cancer therapy. Therefore, we tried to develop a new derivative of BA to enhance its' tumor-suppressive effect without increasing the side effect *in vitro* and *in vivo*.

Previously, BA potently induced protein ubiquitination, including Sp1, to decrease proliferation followed by apoptosis [4, 15], suggesting that BA possibly increases unfolded proteins leading ER stress. In this study, to compare the effect between BA and its derivatives on tumor suppression, apoptosis triggered by various pathways including traditional and ER stress-dependent pathways will be addressed. ER stress, promotes cell survival by adapting to environmental stressors, including the induction of the unfolded protein response, aberrant calcium homeostasis, glucose deprivation, and viral infection [16]. Under persistent stress, the phosphorylation of eukaryotic initiation factor (eIF) 2α phosphorylation is induced, thereby inducing C/EBP homologous protein (CHOP) expression, leading to apoptosis [17]. During tumorigenesis, dysregulation of the unfolded protein response, such as increased protein folding capacity in the ER, can promote cells growth [18]. Several drugs including versipelostatin, bortezomib, and brefeldin A have been designed to prevent the increase in folding capacity, leading to apoptosis [19].

Even though BA was confirmed as a potent anti-cancer drug, the effects of BA and its' derivatives on metastasis is rarely studied. Herein, we not only study the difference between BA and SYK023 in suppressing tumorigenesis, but also metastasis. Metastasis occurs when cancer cells dissociate from primary tumor, migrate through the connective tissue and capillary walls to the circulatory system, and form a tumor at a secondary site [20]. Numerous processes are involved in the alterations to cell shape and migration that promote metastasis, including enhanced integrin expression, protease secretion, modification of integrin/actin linkage proteins, and the coordinated activation of the actin cytoskeleton [20, 21]. Many actin-associated proteins such as tropomyosin and α-actinin are involved in cancer metastasis [22, 23]. Synaptopodin (SYPD), an actin-binding protein, was initially identified as a regulator of the dendritic spine in telencephalic neurons and the foot processes of renal podocytes [24]. Recently, SYPD was shown to regulate synapse plasticity [25]. Furthermore, SYPD also stabilizes actin microfilaments and Rho family proteins, thereby promoting migration [26, 27]. However, the role of SYPD in cancer remains unclear despite its ability to regulate migration and actin remodeling.

In this study, we used two mouse models with either Kras^G12D^- or EGFR^L858R^-induced lung tumor to evaluate the therapeutic efficacy of BA derivatives. SYK023, a derivative of BA, contains stronger inhibitory activity than BA in both lung cancer formations through induction of ER stress-dependent apoptosis and reduced the metastasis of lung cancer by impairing F-actin remodeling.

## RESULTS

### BA derivatives display enhanced selectivity and cytotoxicity than BA on lung cancer *in vitro* and *in vivo*

**Figure 1:**
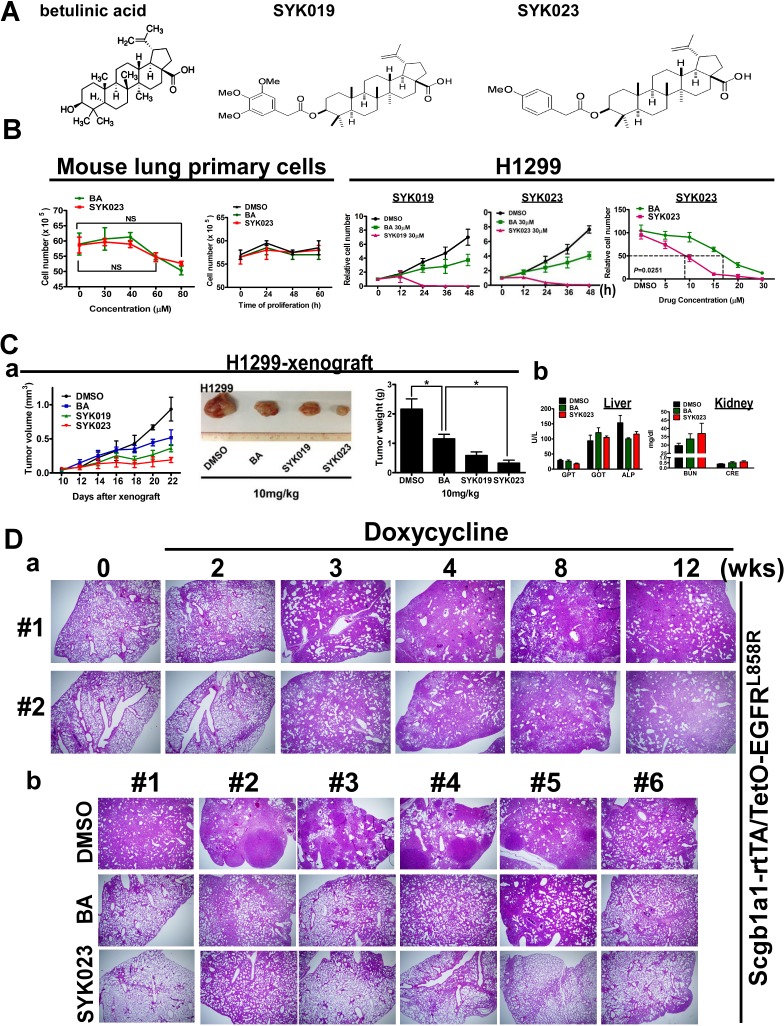
The improved tumor-suppressive effect of BA derivative, SYK023, on lung cancer, *in vitro* and *in vivo* **A**. The chemical structure of BA, SYK019 and SYK023. **B**. Effects of BA, SYK019 and SYK023 on cell proliferation of mouse primary cells and H1299 cells. Cells were treated with the indicated drug, and trypsinized for counting by hemacytometer. Data are expressed as mean±s.e.m, *P*-value is indicated. To compare the survival curves of BA- and SYK023-treated H1299 cells, two-way ANOVA was used. **C**. Effects of BA and derivatives on tumor growth and physiological functions. After 10 days of tumor xenografting (Day 10), the drug (10 mg/kg) was intraperitoneally injected into SCID mice. On Day 25, tumors were exicsed, and serum was collected. **D**. (a). EGFR^L858R^ mice were treated with doxycycline for the indicated period. (b). On the 3rd week, mice were administrated with BA or SYK023 for 9 weeks (10 mg/kg) in the presence of doxycycline treatment, and lungs were excised for HE staining.

Our recent study showed that BA inhibits lung tumor growth *in vitro* and *in vivo* [4]. To identify the compounds that best suppress tumorigenesis, twenty BA derivatives were synthesized and their cytotoxicity in lung cancer cells was evaluated (Supplementary Figure S1). We found that SYK019 and SYK023 (Figure 1A-1C) inhibited tumorigenesis better than BA. Interestingly, neither BA nor SYK023 affected cell survival in normal mouse primary lung cells (Figure 1B, left panel and Supplementary Figure S2).

To evaluate the anti-tumor effect of the BA derivatives *in vivo*, we used xenograft models and transgenic mice that develop spontaneous lung tumors following doxycycline treatment in Scgb1a1-rtTA/TetO-Kras4b^G12D^ and Scgb1a1-rtTA /TetO-EGFR^L858R^ mice. In xenograft models, SYK023 significantly reduced tumor volume and weight (Figure 1C and Supplementary Figure S3). In physiological evaluation, there is no significant difference between normal and lung cancer mice (Table 1). In particular, we also found that BA and SYK023 did not affect the physiological functions of the liver and kidneys by measuring several metabolic enzymes (Figure 1C(c)), including glutamic-pyruvate transaminase (GPT), glutamic-oxaloacetic transaminase (GOT), alkaline phosphatase (ALP), blood urea nitrogen (BUN), and creatinine (CRE) (Table 1).

**Table 1:** The physiological parameters of mice receiving DMSO, BA or SYK023

Mice	Parameters	DMSO (D)	BA	SYK023	D vs BA	D vs SYK
Kras4b^G12D^ (n=8)	ALP (U/L)	233±15.2	197±14.8	182±16.1	*P*=0.1118	*P*=0.0375*
	GOT (U/L)	51.9±7.9	48±2.9	50.9±6.2	*P*=0.6509	*P*=0.9217
	GPT (U/L)	38.4±6.2	30.6±3.2	38.1±2.8	*P*=0.2843	*P*=0.9711
	BUN (mg/dl)	25.5±2	29.6±1.6	26.1±1.7	*P*=0.1279	*P*=0.8141
	CRE (mg/dl)	0.6±0.08	0.5±0.08	0.4±0.09	*P*=0.6160	*P*=0.2158
SCID (n=6)	ALP (U/L)	153.5±24.6	100.3±3.6	116.2±8	*P*=0.0586	*P*=0.1803
	GOT (U/L)	93.8±18.8	121±16.1	104.8±5	*P*=0.2977	*P*=0.5838
	GPT (U/L)	28.8±3.7	26.3±4.2	17.7±2.4	*P*=0.6660	*P*=0.0303*
	BUN (mg/dl)	29.7±1.4	33.7±3.1	36.9±6.2	*P*=0.2703	*P*=0.2774
	CRE (mg/dl)	0.4±0.04	0.5±0.1	0.6±0.1	*P*=0.3293	*P*=0.1284

Data were expressed as mean±s.e.m.

* P<0.05

**Figure 2:**
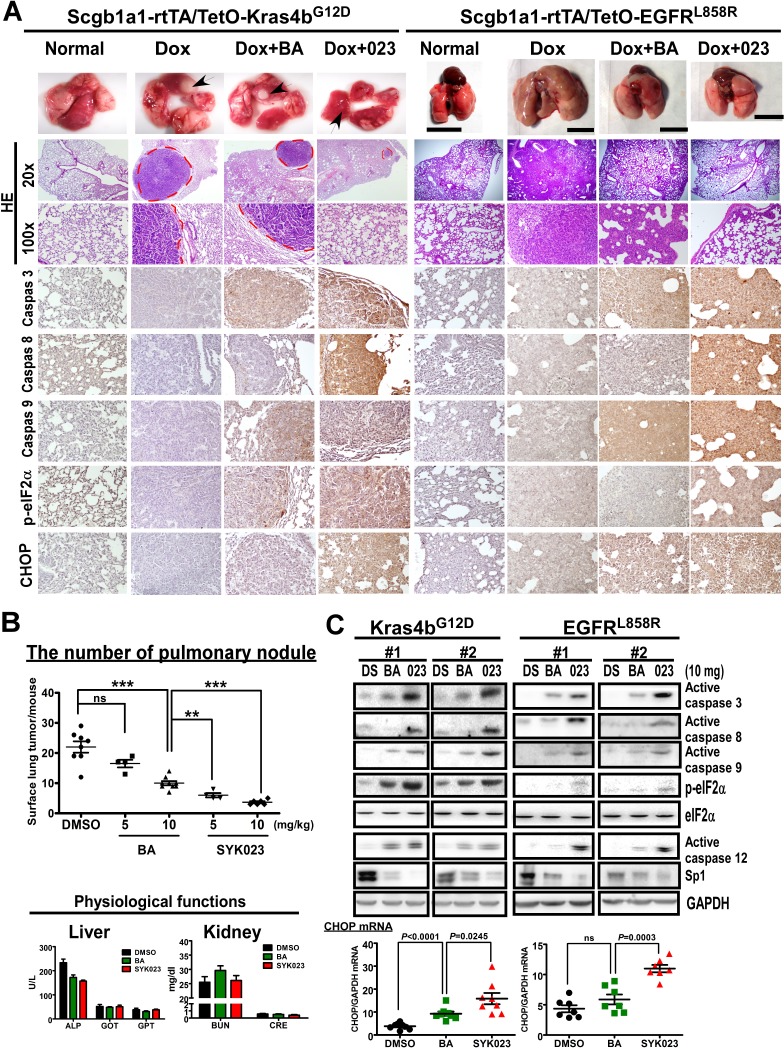
SYK023 activates caspase cascade and induces ER stress *in vivo* **A**. Effects of BA and SYK023 on lung tumor progression in Kras^G12D^ and EGFR^L858R^ transgenic mice. After treatment with BA or SYK023 (10 mg/kg), lungs were excised for histological staining. Scale bar means 1 cm. **B**. The number of tumor nodules on lung surface of Kras^G12D^ mice. The serum was collected for evaluating liver and kidney functions. Data are expressed as mean±s.e.m. *P < 0.05, **P < 0.01 and ***P < 0.001. **C**. The whole tissue extracts of lungs were prepared for Western blotting. The mRNA was subjected to reverse transcription and Q-PCR. Data are expressed as mean±s.e.m, *P*-value is indicated.

We next assessed the effect of the BA derivatives in the transgenic lung cancer models. After confirming that doxycycline-induced lung cancer formation in EGFR^L858R^ mice, we treated the mice with BA or SYK023 starting at week 3, for 9 weeks total. While BA had a slight anti-tumorigenic effect, SYK023 potently blocked lung cancer progression (Figures 1D, 2A and Supplementary Figure S3). Compared to BA, SYK023 also inhibited lung tumorigenesis in Kras4b^G12D^ mice, without affecting liver and kidney functions (Figure 2B). In contrast, SYK019 had adverse health effects in mice because the mortality of SYK019-injected mice was higher than that of other drug-treated mice (Supplementary Table S1). In addition, SYK019 was not effective against lung tumors when compared to SYK023 (Figure 1C(a-b)). Therefore, we did not continue to study the effect of SYK019 on lung cancer. Histological analysis of Kras^G12D^ and EGFR^L858R^ mice showed that the extent of tumor formation decreased in SYK023- treated mice compared to that in BA-treated mice (Figure 2A, HE staining).

We next assessed how SYK023 inhibited lung tumor growth. As shown in Figure 2A and 2C, SYK023 activated the apoptotic pathway, as indicated by the increase in active caspase 3, caspase 8 and caspase 9. Furthermore, SYK023 also induced ER stress, as indicated by the phosphorylation of eIF2α, the increase in active caspase 12 and the transcriptional upregulation of CHOP. Interestingly, Sp1 was also decreased by BA and SYK023 in both lung cancer models. These effects were more evident in the SYK023 than in the BA treated mice, as BA did not induce apoptosis in the lung tumors efficiently, and did not induce ER stress in the EGFR^L858R^ mice. Taken together, our data indicate that SYK023 has improved tumor-suppressive activity compared to BA due to its ability to activate caspases and induce ER stress.

### SYK023-induced ER stress contributes to lung cancer cell apoptosis and cell cycle progression

**Figure 3:**
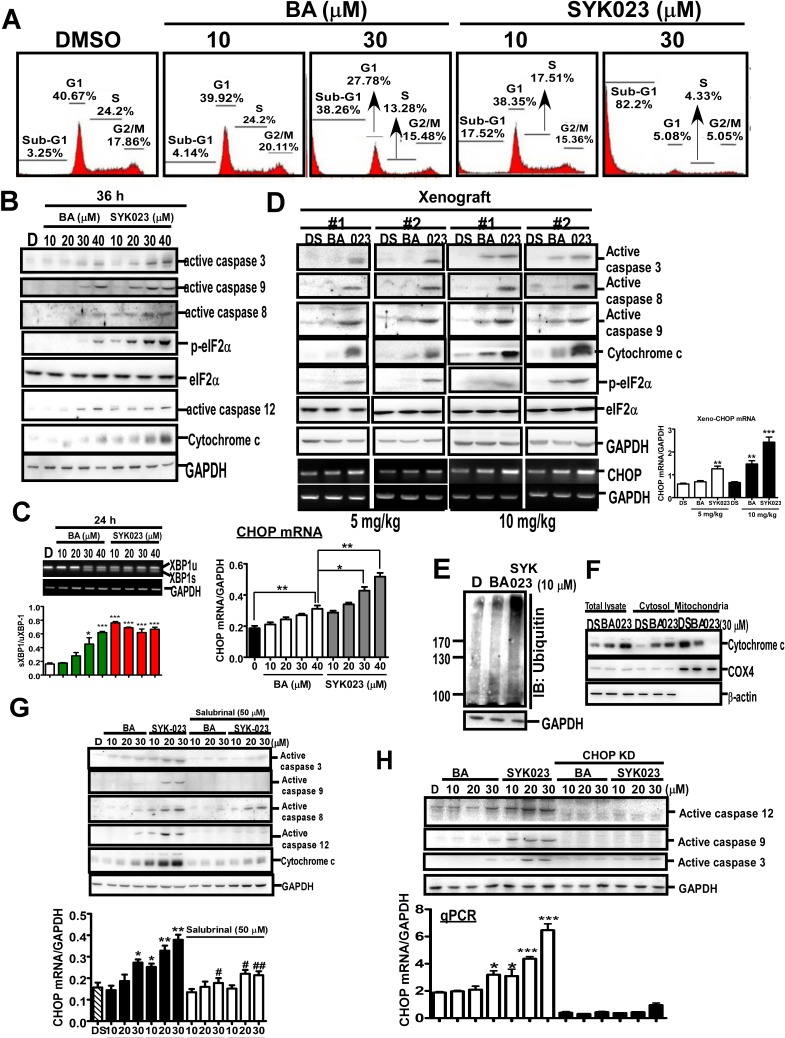
SYK023 induces ER stress-dependent apoptosis in lung cancer **A**. After treatment with BA or SYK023, H1299 cells were subjected to flow cytometry. **B**. Cells were harvested for Western blotting. **C**. Upper, the splicing of XBP-1 was analyzed by PCR following agarose electrophoresis. Lower panel, The quantitated result of XBP-1 splicing. Spliced form was normalized with unspliced form. **D**. Whole tissue extracts of tumors from H1299-xenografted SCID mice were analyzed by Western blotting. **E**. After treatment for 36 h, H1299 cells were harvested for Western blotting. **F**. Cytosolic and mitochondrial fractions were prepared for Western blotting. **G**. After pre-treatment with SAL (50 μM) for 2 h, H1299 cells were treated with BA or SYK023 for 36 h. Cell lysates were analyzed by Western blotting and qPCR. **H**. After CHOP knockdown, cells were treated with BA or SYK023 for 36 h. Cell lysates were analyzed by Western blotting and qPCR.

We next studied the detailed molecular mechanism of SYK023-induced apoptosis and ER stress (Figure 3). In H1299 cells, 10 μM SYK023, not BA, increased the expression of apoptotic markers, annexin V staining and the percentage of cells in the sub-G1 phase (Figure 3A and Supplementary Figure S4A). Moreover, SYK023 increased eIF2α phosphorylation, active caspase 12, X-box-binding protein 1 (XBP1) splicing, and CHOP transcription in H1299 cells and xenograft mice, indicating that SYK023 induces apoptosis and ER stress *in vitro* and *in vivo* (Figure 3B-3D). Furthermore, SYK023 (10 μM), not BA, increased total protein ubiquitination (Figure 3E), suggesting that the accumulated misfolded proteins are degraded by the ER-associated degradation pathway during the process of ER stress. ER stress was previously shown to induce apoptosis through the impairment of mitochondrial membrane potential, resulting in the release of cytochrome c. SYK023 strongly induced the release of cytochrome c from the mitochondria to the cytosol (Figure 3F), suggesting that SYK023-induced apoptosis is partially dependent on ER stress. To explore whether the apoptosis was mediated by ER stress, the eIF2α inhibitor, salubrinal (SAL), was used. We initially confirmed that SAL is an eIF2α dephosphorylation inhibitor in Supplementary Figure S4B [28]. In Supplementary Figure S4C, SAL did not induce ER stress and apoptosis, characterized by active caspase 12 and caspase 3, respectively, in H1299 cells. Therefore, pretreatment with SAL for 2 h does not influence cell survival in subsequent experiments. We also further confirmed that SAL significantly prevented tunicamycin (TM)-induced ER stress (Supplementary Figure S4D). By the way, both the reactive oxygen species (ROS) scavenger, *N*-acetyl-L-cysteine (NAC) [7], and SAL partially inhibited BA- and SYK023-induced cell death (Supplementary Figure S4E). In particular, when ER stress was inhibited by SAL (Figure 3G) or CHOP knockdown (Figure 3H), BA and SYK023 failed to induce the intrinsic apoptotic pathway. These results suggest that SYK023-induced ER stress increases apoptosis of lung cancer cells.

**Figure 4:**
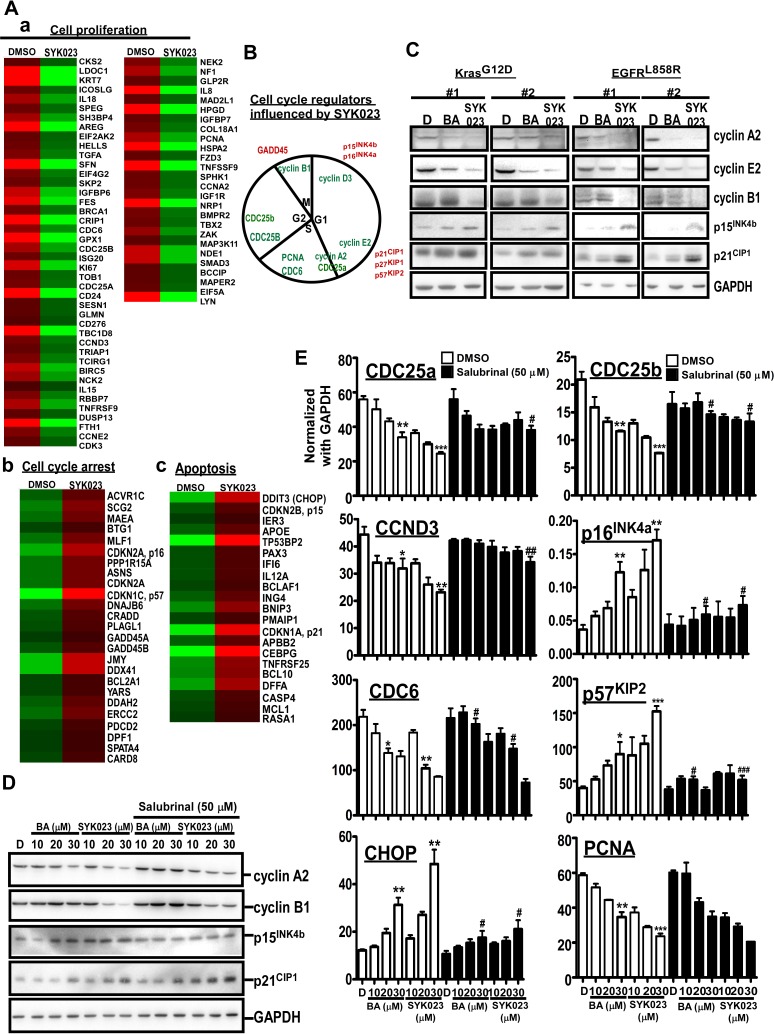
Gene expression profile altered by SYK023-induced ER stress **A**. Micorarray analysis of DMSO- and SYK023 (20 μM)-treated cells. **B**. Cell cycle regulators affected by SYK023 were summarized. Red is upregulation, and green is downregulation. **C**. Upper panel, lysates of lungs were analyzed by Western blotting. Lower panel is quantitated results. **D**. Upper panel, whole cell lysates of H1299 were prepared for Western blotting. Lower panel panel is quantitated results. E. The mRNA was analyzed by Q-PCR using indicated primers. Data are expressed as mean±s.e.m. Compared with DMSO without SAL: *P < 0.05, **P < 0.01 and ***P < 0.001. The comparison between groups with or without SAL: #*P* < 0.05, #*P* < 0.01 and #*P* < 0.001.

We next assessed how SYK023-induced ER stress affected cell survival at the molecular level. Global gene expression profiles of lung cancer cells with or without SYK023 treatment were analyzed by cDNA microarray (Figure 4A-4B). Interestingly, genes involved in cell proliferation were downregulated following SYK023 treatment. In contrast, genes related to cell cycle arrest and apoptosis were upregulated (Figure 4A-4B). In mice with lung cancer, both of BA and SYK023 obviously decreased the levels of cyclin A2 and cyclin E2, whereas those of p15^INK4b^ and p21^CIP1^ increased (Figure 4C). Further, microarray analysis identified that the cyclin B1-interacting E3 ligase was upregulated by SYK023 (data not shown), suggesting that cyclin B1 levels may be decreased. Accordingly, SYK023-treated mice exhibited lower expression of cyclin B1; however, BA did not affect cyclin B1 expression. This result suggests that the downregulation of cyclin B1 may regulate the enhanced anti-tumor effect of SYK023 versus BA. Furthermore, the downregulation of cyclin A2, cyclin B1, CDC25a, CDC25b, CCND3, and CDC6 by SYK023 and the upregulation of p15^INK4b^, p16^INK4a^, p57^KIP2^, and CHOP by SYK023 were reversed by blocking ER stress (Figure 4D-4E). However, the upregulation of p21^CIP1^ and the downregulation of proliferating cell nuclear antigen (PCNA) were not dependent on ER stress, suggesting that SYK023 inhibits cell cycle progression through ER stress-dependent and -independent pathways.

### SYK023 inhibits lung tumor metastasis through the inhibition of F-actin polymerization

**Figure 5:**
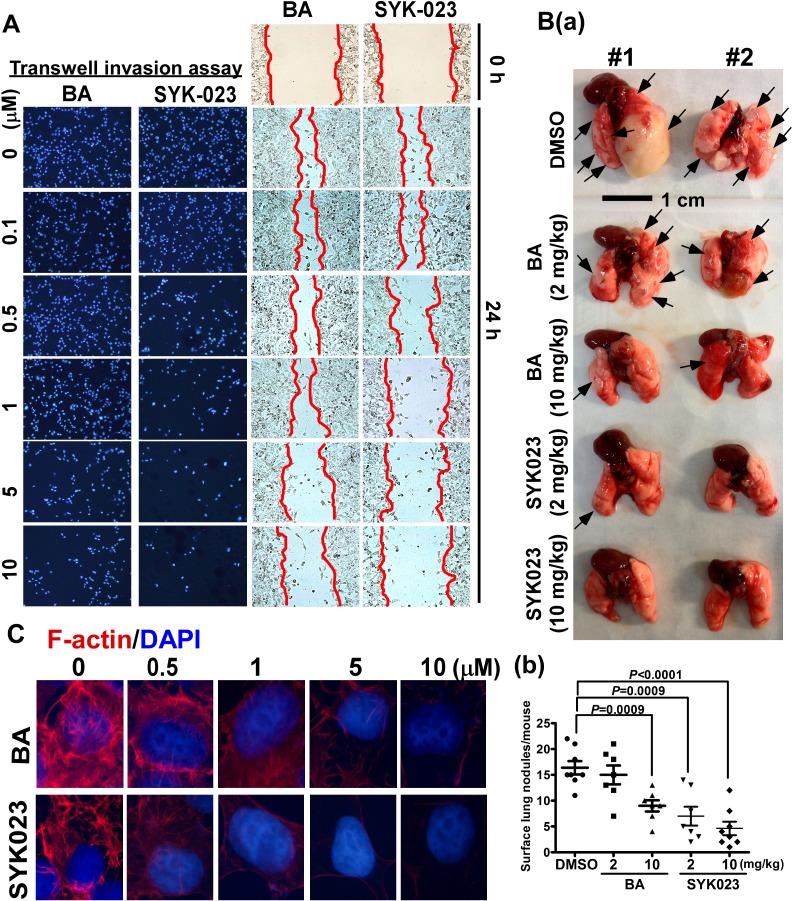
Effects of BA and SYK023 on lung tumor metastasis *in vitro* and *in vivo* **A**. After BA and SYK023 treatment for 36 h, H1299 cells were subjected to transwell invasion and wound-healing assay. **B**. Representative images of lungs from SCID mice with metastasis (a). The number of surface lung tumors. Data are expressed as mean±s.e.m, *P*-value is indicated (b). **C**. H1299 cells on the coverslip were treated with the indicated drug, and subjected to immunoflurescent staining for F-actin and DAPI (1000x).

Using both transwell invasion and wound-healing assays, we found that SYK023 (0.5 μM) obviously inhibited migration without causing cell death, as compared to BA (5 μM) (Figure 5A and Supplementary Figure S5). Furthermore, compared to BA, SYK023 (2 mg/kg) significantly inhibited metastasis *in vivo* (Figure 5B). Because F-actin regulates cytoskeletal dynamics to promote migration, we assessed whether BA and SYK023 affected F-actin remodeling. We found that F-actin polymerization was attenuated by SYK023 (0.5 μM) or by BA (5 μM) (Figure 5C and Supplementary Figure S6). Strikingly, increased doses of SYK023 both blocked F-actin polymerization and decreased the F-actin levels. These results suggest that SYK023 inhibits migration by inhibiting F-actin polymerization.

**Figure 6:**
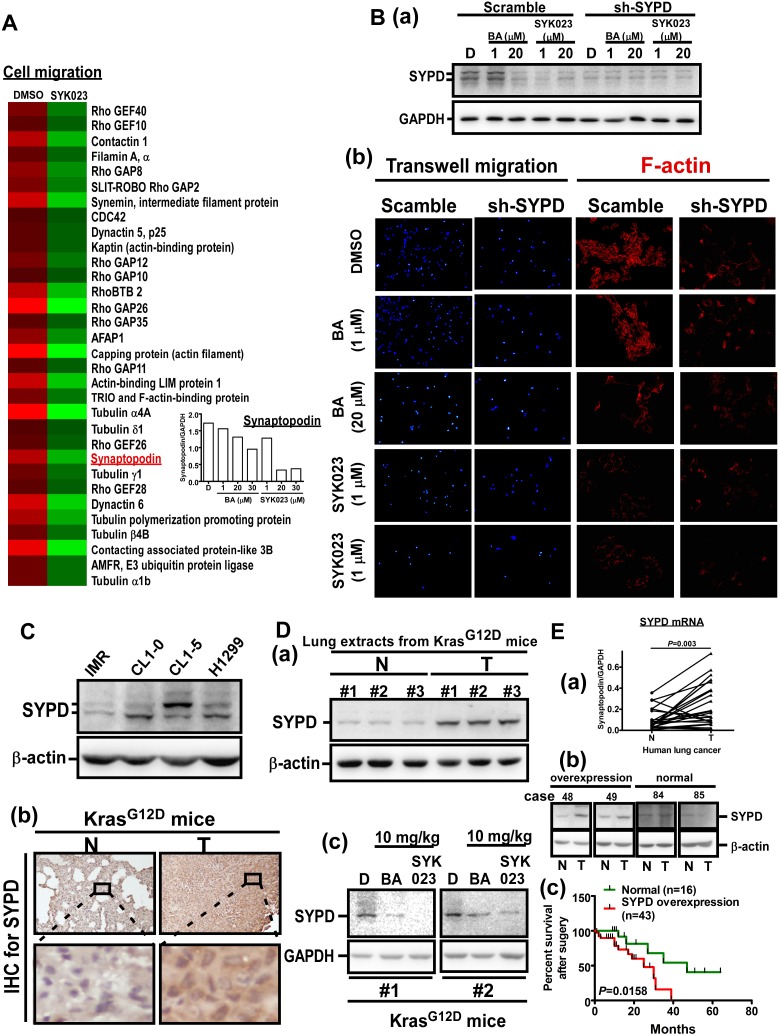
SYK023-targeted SYPD is a novel biomarker of lung cancer **A**. Micorarray analysis of DMSO- and SYK023-treated cells. Q-PCR for SYPD. **B**. After transfection with scramble or SYPD shRNA for 48 h, cells were trypsinzed for Western blotting (a), or seeded onto coverslips for immunofluorescence of F-actin (200x) (b). **C**. The cell lysates were analyzed by Western blotting. **D**. Lung tissues from Kras^G12D^ mice were analyzed by Western blotting (a and c) and immunohistochemistry for SYPD (b). **E**. The mRNA level of normal and cancer from lung cancer patients was analyzed by Q-PCR. Data are expressed as mean±s.e.m, *P*-value is indicated (a). The representative images of overexpression and normal expression of SYPD protein in human lung cancer (b). The survival rate of 59 lung cancer patients according to the level of SYPD (c).

Based on these data, we investigated the effect of SYK023 on signaling pathways that mediate F-actin remodeling. The phosphorylation of FAK, Src, Akt and mTOR, all of which promote F-actin polymerization [29-31], was reduced by SYK023 (Supplementary Figure S7A). In addition, we found that SYK023 (5 μM) effectively decreased the expression of some proteins that promote lung tumor formation (Supplementary Figure 7B), including N-cadherin, β-catenin, vimentin, and c-myc [32-35]. These findings support the notion that low doses of SYK023 can impair F-actin and oncoprotein expression, without inducing apoptosis. Similar effects were not observed with BA. Moreover, microarray analysis, which functionally grouped using the Gene Set Enrichment Analysis website (Figure 6A), revealed that several genes related to migration were decreased by SYK023, including Rho family proteins, contactin-1, intermediate filament proteins and tubulins [36-39]. These data suggest that SYK023 inhibits migration by dysregulating cytoskeletal structure.

Interestingly, the actin-binding protein SYPD was also downregulated by SYK023. SYPD, is known to regulate cell migration and to stabilize RhoA in podocytes. [27]. Importantly, SYPD has never been studied in cancer. We confirmed that SYPD knockdown blocked F-actin polymerization and expression (Supplementary Figure S8). Moreover, SYPD expression was decreased by BA (20 μM) and SYK023 (1 μM) (Figure 6B). Furthermore, in addition to attenuating migration, SYPD knockdown prevented the inhibitory effect of BA and SYK023 on migration and F-actin remodeling (Figure 6B). In conclusion, many proteins including SYPD, are involved in F-actin polymerization and are down regulated following SYK023 treatment.

### Overexpression of SYPD is a biomarker of lung cancer to predict poor prognosis

Since the role of SYPD in cancer is unknown, we assessed whether SYPD is important for lung cancer progression. Compared with IMR cells, a normal pulmonary fibroblast cell line, the protein level of SYPD was increased in lung cancer cells, including CL1-0, CL1-5 and A549 cells (Figure 6C). In Kras^G12D^ mice (Figure 6D), SYPD expression was upregulated in lung tumors. As expected, SYPD was reduced in the lungs of BA- and SYK023-treated Kras^G12D^ mice. To establish the clinical relevance, we analyzed the SYPD level in normal and tumor tissues from lung cancer patients that underwent surgery (Figure 6E and Supplementary Figure S9). In 59 patients, 43 exhibited higher SYPD expression in tumor tissue compared to normal tissue. In addition, the mRNA level of SYPD was significantly higher in lung tumors. Importantly, we found that SYPD overexpression correlated with poor prognosis, suggesting that SYPD, an actin-binding protein, participates in lung cancer malignancy.

## DISCUSSION

In this study, we screened a new BA derivative, SYK023, with higher tumor-suppressive activities and lower side-effect to normal lung normal cells compared to BA and other BA derivatives. Although several other BA derivatives have been studied, the *in vivo* evidence of anti-tumor activity is still unclear. Herein we not only used more *in vivo* tool, spontaneously lung cancer animal models to evaluate the anti-tumorigenesis activity of SYK023, but first time found SYK023 with anti-metastasis activity through affecting F-actin polymerization.

BA has been shown to have anti-virus, anti-inflammation and anti-cancer activity [1]. BA has been shown to inhibit a variety of cancers, including melanoma, head and neck, colon, breast, neuroectodermal tumors, prostate, renal, ovarian, lung, and cervical cancers, as well as leukemia, as assessed in cancer cell lines or xenograft mouse models [1, 3-7]. Furthermore, we recently used xenograft mouse models and a Kras^G12D^-induced lung cancer mouse model to assess the effect of BA, and found that it suppresses tumorigenesis by inhibiting cell cycle progression [4]. Collectively, previous studies indicate that BA inhibits tumorigenesis by promoting mitochondria potential-induced cell apoptosis and the inhibition of cellular proliferation [1]. In this study, we studied the effect of 27 BA derivatives on lung cancer cell proliferation. Several derivatives including SYK010, SYK019 and SYK023 were more cytotoxic in lung cancer cells than BA (Supplementary Figure S1). Further testing revealed that SYK023 had fewer side effects in primary lung cells and mice than BA, SYK010 and SYK019 (Supplementary Table S1). Therefore, we assessed the molecular mechanisms by which SYK023 inhibited lung tumorigenesis. Previous studies indicated that BA inhibits the proliferation of cancer cells through inhibiting cell cycle progression and triggering the intrinsic apoptotic pathway [1]. In this study, we found that SYK023 not only induced cell cycle arrest and extrinsic apoptotic pathway, but also induced ER stress-dependent intrinsic apoptosis. Microarray analysis revealed that the mRNA level of CCNB1 was not altered following SYK023 treatment; however, CCNB1 interacting protein 1 (CCNB1IP1), an E3 ubiquitin ligase targeting cyclin B1 [40], was increased (data not shown), suggesting that SYK023 may affect more anti-tumor pathways than BA to inhibit cancer cell proliferation. We then hypothesized that SYK023 may enhance the E3 ligase activity of CCNB1IP1 to degrade cyclins. In addition, SYK023 may also induces the expression of other unknown E3 ligases, based on the strong ubiquitinated signal in SYK023-treated cells (Figure 3D). Simultaneously, this signal may be attributed to the accumulation of unfolded proteins leading to ER stress or the inhibition of multiple deubiquitinases in cancer cells [15]. The other nature compound, oplopantriol, exhibits activity similar to that of SYK023, inducing the accumulation of ubiquitinated proteins and ER stress [41]. In the past, several well-known anti-cancer drugs exert their tumor killing effects through the induction of ER stress, including bortezomim, brefeldin A, curcumin, celecoxib, and heat shock protein 90 inhibitors [42].

To confirm whether SAL increases eIF2α phosphorylation [28], we treated H1299 cells for the indicated period. As shown in Supplementary Figure S4B, SAL did not affect p-eIF2α level until 16^th^ h. In our experiments which combines SYK023 and SAL, cells were pre-treated with SAL for only 2 h. Therefore, SAL-only should not affect eIF2α phosphorylation when cells were treated with BA or SYK023. When we performed experiments related to SAL, time of SAL treatment is only 2h. We thought this treatment time do not affect cell survival. However, to test whether SAL induces ER stress or apoptosis, we treated H1299 cells with SAL (50 μM) for 24h. As shown in Supplementary Figure S4C, SAL did not decrease proliferation until 36h, indicating that SAL did not affect cell survival during the period of SYK023 treatment. Compared with tunicamycin treatment for 24h, neither caspase 12 nor caspase 3 cleavage was not induced by SAL. CHOP transcription was not induced by SAL. Based on this data, we make sure that treatment of H1299 cells with SAL for 24h did not induce ER stress or apoptosis. Because we cannot explain why combination of SYK023 with SAL decreases eIF2α phosphorylation. We used caspase 12 and CHOP mRNA as the ER stress marker instead of p-eIF2α (Figure 3G) [43, 44]. However, SAL still attenuated SYK023-induced caspase 12 cleavage and CHOP expression (Figure 3G). In microarray results (Figure 4A(b)), PPP151A/GADD34, which was shown to dephosphorylate eIF2α phosphorylation [45], was increased by SYK023. The inhibitory effect of SAL on eIF2α phosphorylation maybe thus antagonized by SYK023 through GADD34. In Figure 3G, SAL prevented SYK023-induced apoptosis, suggesting that SYK023 causes cell death through inducing ER stress. Moreover, SYK023-mediated cytoskeleton impairment also contributes to prevent metastasis activity of lung cancer. In Figure 5B, 2 mg/kg of SYK023, not BA, significantly inhibited metastasis *in vivo*, suggesting that, in this study, we screen a BA derivative, SYK023, contains higher inhibited activities of tumorigenesis and metastasis and lower side-effect in normal cells. In addition, previous studies also indicated that BA decreases Sp1 levels in prostate and lung cancers [3, 23].

In studying the effects of SYK023 and BA in the Kras^G12D^- and EGFR^L858R^-induced lung cancer mouse models, we found that SYK023 dramatically inhibited lung cancer nodule formation in both systems (Figures 1D, 2A and 2B). However, BA inhibited Kras^G12D^-induced lung cancer formation significantly, but had the slightly inhibitory effect on EGFR^L858R^-induced lung cancer (Figures 1D, 2A and 2B). Furthermore, we also found that Sp1 was dramatically accumulated in the lung tissue of Kras^G12D^-induced lung cancer mice [46] but not found in EGFR^L858R^-induced lung cancer mice. According to the results of this study, we hypothesized that inhibition of lung cancer growth by BA is through Sp1 degradation, and in contrast, that of SYK023 is through both Sp1 degradation and ER stress. Our previous study indicated that there is an inverse correlation between Sp1 level and prognosis in clinical lung cancer patients, suggesting that SYK023, but not BA, may be the better therapeutic option for lung cancer, particularly for that without Sp1 accumulation which is associated with poor prognosis [46].

The anti-tumor activity of BA *in vivo* is much poorer than that of SYK023, especially in EGFR^L858R^ mice (Figure 1), suggesting that, compared to SYK023, BA requires much higher dose for tumor therapy *in vivo*. This increases the possibility of side effects. Although BA did not affect the survival of normal cells, it still cause the death of mice during the period of treatment (Supplementary Table S1). Herein, the number of dead mice by SYK023 was obviously fewer than that by BA, suggesting that the tolerance of mice to SYK023 is much better than that to BA. SYK023 highly improved the cytotoxicity of BA in tumor cells without increasing the occurrence of side effect. This enhances the worth and possibility of the BA derivative, SYK023, in clinical usage. Based on results of Figures 2 and 5, the working dose of SYK023 and BA is 2-5 mg/kg and 10-20 mg/kg, respectively. For a 70 kg of man with lung cancer, the pastille form of SYK023 is 140-350 mg/tab, which is similar with that of gefitinib (250 mg/tab) and is easily received by patients; that of BA requires 700-1400 mg/tab. In addition, in Figures 2C and 3D, SYK023, not BA, potently induced extrinsic apoptosis which was not dependent on ER stress (Figure 3G).

In this study, we established that BA and SYK023 can inhibit the metastasis of lung cancer. Low dose SYK023 (0.5 μM) significantly inhibited lung cancer metastasis (Figure 5). Gene expression profile analysis revealed that several metastasis-related genes, including SYPD, were decreased following SYK023 treatment (Figure 6), suggesting that SYK023 might affect the expression of many actin polymerization-related genes to inhibit lung cancer cell migration. However, how SYK023 inhibits gene expression remains unclear. Our analysis also revealed that SYK023 decreased the phosphorylation of Src, FAK, Akt and mTOR (Supplementary Figure S7), all of which are involved in cell survival, apoptotic pathways, and actin polymerization in lung cancer [47, 48]. In Figure 4D, SAL partially reversed the decrease of cyclin expression by SYK023, suggesting that SYK023 reduces cyclin through ER stress-dependent and -independent pathway. In addition to inducing ER stress, SYK023 (1 μM) also destroyed cytoskeleton. This is not contributed by ER stress. The impairment of cytoskeleton also contributes to the alteration of gene profile. Some genes dysregulated by SYK023 are through ER stress, some are independent of ER stress. BA-induced ER stress maybe not enough to drive cells for apoptosis. It is possibly reversible. At 20-30 μM, the cytotoxicity of BA maybe restricted in ER stress-induced mitochondria dependent apoptosis. Therefore, BA-induced the alteration of gene expression was prevented by inhibiting ER stress.

Metastasis is the most serious cancer-related problem because it is not easily diagnosed in lung cancer and often occurs before cancer has been diagnosed. Treatments for lung cancer, including radiation, chemotherapy, surgery, and targeted therapy, are options for treating lung cancer that has metastasized to the liver, brain, and bone. Testing can be performed to distinguish if the cancer expresses mutations that have a targeted therapy. For example, if a patient has an EGFR mutation, erlotinib (Tarceva) and gefitinib (Iressa) can be used [49]. Alternatively, the presence of an anaplastic lymphoma kinase (ALK) gene abnormality allows for treatment with crizotinib (Xalkori) [50]. In addition, other specific abnormalities, such as RET, ROS-1, and KRAS mutations are being studied to develop specific inhibitors. In this study, SYK023 was used in Kras^G12D^- and EGFR^L858R^-induced lung cancer to inhibit cancer formation (Figure 1), suggesting that SYK023 is useful for treating lung cancer having oncogene mutation. Furthermore, various metastasis-related experiments were performed *in vitro* and *in vivo* to show that SYK023 not only decreases cancer cell proliferation but also inhibits lung cancer metastasis at low doses. In addition to SYPD, several cytoskeletal proteins known to regulate migration or invasion were also decreased by SYK023, including Rho family proteins [51, 52], contactin-1 [53], filamin A [54], synemin [38], capping protein [55], actin-binding LIM protein 1 [56], TRIO, [57] and tubulin family [52] (Figure 6A). Based on these findings, SYK023 may be a viable treatment for lung cancer metastasis, either alone or in combination, without causing damage to normal cells.

## MATERIALS AND METHODS

### Cell culture and chemical compounds

H1299 and A549 lung cancer cell lines were purchased from American Type Culture Collection (Manassas, VA, USA). The CL-series cell lines were provided by Dr. Pan-Chyr Yang (National Taiwan University, Taipei, Taiwan). These cells were cultured in Dulbecco's modified Eagle's medium (DMEM; Invitrogen, Carlsbad, CA, USA) supplemented with 10% FBS (Invitrogen), 100 mg/ml streptomycin sulfate and 100 U/ml penicillin G sodium at 37°C in 5% CO_2_. Normal mouse primary lung cells were isolated from lungs homogenized by using 0.5% collagenase (Sigma-Aldrich, St. Louis, MO, USA) and 0.05% trypsin (Biological Industries, Kibbutz Beit Haemek, Israel) at 37°C for 1 h. After centrifuging at 1200x g for 20 min, and filtering, cells were resuspended by DMEM-10% FBS, and seeded onto 6-well plates. BA was purchased from Sigma-Aldrich. Compounds SYK001, 002, 003, 004, 005, 006 and 009 were synthesized according to the previous study [58]. The eIF2α inhibitor, salubrinal, was purchased from Biovision (Milpitas, CA, USA). BA, the derivatives and salubrinal were dissolved in dimethyl sulfoxide (DMSO; Sigma-Aldrich).

### Transfection

H1299 cells were transfected with indicated shRNA with lipofectamine 2000 (Invitrogen) according to the manufacture in Opti-MEM for 9 h, and incubated for additional 16 h for subsequent experiments.

### The synthesis of other SYK compounds

The synthetic protocol of SYK compounds was described in *Supplementary Materials and Methods*.

### General experimental procedures for drug synthesis

^1^H NMR spectra were measured on a 400 MHz Varians Gemini 2000 spectrometer using TMS as internal standard. NMR spectra were recorded in CDCl_3_. Mass spectra were recorded on a Shimadzu LC-MS2010 instrument. Analytical TLC was performed on pre-coated silica gel GF plates from Merck, Inc (Darmstadt, Germany). Medium-pressure column chromatography was performed using a CombiFlash^®^ Companion system from ISCO, Inc (Columbus, Ohio, USA). 1-naphthoic acid and 4-benzyloxyphenylacetic acid were purchased from Acros Organics (Geel, Belgium) and TCI Co., Ltd. (Tokyo, Japan), respectively. All other chemicals purchased from Sigma-Aldrich.

### 3-O-(4-Methoxyphenylacetyl)betulinic acid (SYK023)

A solution of 4-methoxyphenylacetic acid (94.8 mg, 0.57 mmol) in CH_2_Cl_2_ (4.5 mL) and Et_3_N (0.5 mL) was treated with EDCI (86.6 mg, 0.56 mmol) and DMAP (57.6 mg, 0.47 mmol), and 20 min later betulinic acid (49.4 mg, 0.11 mmol) was added. The reaction mixture was stirred overnight before water was added. The mixture was extracted with CH_2_Cl_2_, washed with brine, and dried over MgSO_4_. The solvent was removed, and the residue was submitted to Medium-pressure column chromatography with *n*-hexane/EtOAc to give SYK023 (11.8 mg, 18%) as white powder; ^1^H NMR (400 MHz, CDCl_3_): δ 7.29 (2H, d, *J* = 8.8 Hz, Ar-H-2, 6), 6.85 (2H, d, *J* = 8.8 Hz, Ar-H-3, 5), 4.73 (1H, d, *J* = 1.5 Hz, H-29), 4.61 (1H, br s, H-29), 4.45 (1H, dd, *J* = 5.8, 10.8 Hz, H-3), 3.79 (3H, s, OCH_3_), 3.54 (2H, s, OCOCH_2_Ph), 3.00 (1H, dt, *J* = 4.9, 10.8 Hz, H-19), 1.69 (3H, s, H-30), 0.96, 0.92, 0.83, 0.77, and 0.74 (3H each, s, 5 × CH_3_); MS *m/z*: 603.45 (M – H)^−^. This compound, SYK023, was dissolved in 100% DMSO for all the experiments here.

### Animal experiments

The experiments related with animals were approved by the Institutional Animal Care and Use Committee at National Cheng Kung University (NCKU). Scgb1a1-rtTA/TetO-Kras4b^G12D^ transgenic mice were generated as described previously [46]. Both of Scgb1a1-rtTA and TetO-EGFR^L858R^ mice were purchased from Jackson Lab (Bar Harbor, MA, USA). After breeding, Scgb1a1-rtTA/TetO-EGFR^L858R^ mice were used to study lung cancer. To induce lung cancer progression, two month-old of transgenic mice were orally administrated with doxycycline dissolved in RO water (0.5 g/liter). For drug administration, Scgb1a1-rtTA/TetO-Kras4b^G12D^ mice receiving doxycycline for 3 months were injected intraperitoneally with BA or its derivatives (once/3 days) for 2 months in the presence of doxycycline administration. Scgb1a1-rtTA/TetO-EGFR^L858R^ mice receiving doxycycline for 3 weeks were injected with BA or its derivatives (10 mg/kg, once/3 days) for 9 weeks.

To establish the xenograft model, male athymic BALB/c severe combined immunodeficiency (SCID) mice (8 week-old) were purchased from the Animal Center of NCKU. H1299 cells (1 × 10^6^) in 100 μl PBS were injected into the back of mice. After 10 days mice with obvious tumor formation were injected intraperitoneally with BA or its derivatives (once/3 days) for 15 days. During the period of 15 days, tumor size was measured once/2 days according to the previous study [4].

For metastasis assay *in vivo*, on Day 1, CL1-5 cells (1 × 10^6^) in 100 μl of PBS were injected into the right lateral tail vein of male athymic BALB/c nude mice (8 week-old). Drugs were injected intraperitoneally into mice once/3 days until Day 45. After sacrificing, lungs were excised for counting tumor nodules.

### Physiological evaluation

After sacrificing, blood of mice were collected and centrifuged at 12000 rpm for 30 min at 4°C. The supernatant representing serum was analyzed by FUJI DRI-CHEM 4000i system (FUJIFILM Corporation, Tokyo, Japan) to measure indicated physiological parameters. The values of BA- or SYK023-treated group were compared with DMSO-treated group (DMSO), whose lung tumors spontaneously develop. The values of normal mice which did not develop lung tumor with or without DMSO injection were also shown in Table 1.

### Western blotting

The process was performed according to the previous study [46]. After blocking by 5% non-fat milk, PVDF membrane (Millipore, Bedford, MA, USA) was incubated with the primary antibody which was listed in *Supplementary Materials and Methods*. at 4°C overnight. After incubation with the anti-rabbit or anti-mouse IgG conjugated with horseradish peroxidase (Millipore) for 1 h at room temperature, signals were detected using chemiluminescence Alpha Innotech detection system (Alpha Innotech Corp., San Leandro, CA, USA).

### Preparation of mitochondria and cytosolic fractions

The procedure of this experiment was performed using Mitochondria Isolation Kit (Thermo Scientific Pierce, Rockford, IL, USA) according to the manufacture. Briefly, cells were harvested in Reagent A, and mixed with Reagent B. After mixing with Reagent C, cell suspension was centrifuge at 700 × g for 10 min at 4°C. Subsequently, the mitochondria fraction was pelleted by centrifuging supernatant at 12000 × g for 15 min at 4°C, and the supernatant represented the cytosolic fraction. The pellet was washed once by Reagent C, and mixed with sample buffer for Western blotting analysis.

### Immunohistochemical staining

Briefly, dewaxed histological slides were stained using the indicated antibody at room temperature for 1 h. The immunoactivity was detected using a Vectastain ABC kit (Vector Laboratories, Burlingame, CA, USA), and photographed by the microscope (Olympus, Melville, NY, USA)

### Immunofluorescence

After blocking with 3% bovine serum albumin (BSA), cells on the coverslip were stained with phalloidin 568 (Invitrogen) to detect F-actin at room temperature for 1 h. Stained coverslips were mounted with 90% glycerol containing DAPI (Invitrogen), and photographed under the immunofluorescent microscope (Olympus).

### Quantitative (Q)-PCR

The cDNA (1 ng) was subjected to Q-PCR using SyBRGreen (Takara

Bio, Shiga, Japan) by CFX96 REAL-TIME PCR DET SYS (Bio-Rad Laboratories,

Hercules, CA, USA). The primers were listed in Supplementary Table S2.

### *In vitro* transwell invasion assay

The experimental procedure was described previously [46].

### Wound-healing assay

We performed the same procedure with the previous study [46].

### Human specimens of lung cancer

The procedure of collecting human specimens conformed to the human ethics and was approved by the Clinical Research Ethics Committee at NCKU Medical Center.

### Statistical analysis

Student's *t* test was used to compare the difference between two groups. Two-way analysis of variance (ANOVA) was used to compare the increasing cytotoxic effect of BA and SYK023 on H1299 cells. Kaplan–Meier method was used to calculate survival curves and survival rate was compared according to log-rank test.

## SUPPLEMENTARY MATERIALS, FIGURES, TABLES


