# Correction: A novel derivative of betulinic acid, SYK023, suppresses lung cancer growth and malignancy

**DOI:** 10.18632/oncotarget.27297

**Published:** 2019-11-05

**Authors:** Tsung-I Hsu, Ying-Jung Chen, Chia-Yang Hung, Yi-Chang Wang, Sin-Jin Lin, Wu-Chou Su, Ming-Derg Lai, Sang-Yong Kim, Qiang Wang, Keduo Qian, Masuo Goto, Yu Zhao, Yoshiki Kashiwada, Kuo-Hsiung Lee, Wen-Chang Chang, Jan-Jong Hung

**Affiliations:** ^1^ Center for Infection Disease and Signal Research, College of Medicine, National Cheng Kung University, Tainan, Taiwan; ^2^ Institute of Bioinformatics and Biosignal Transduction, College of Bioscience and Biotechnology, National Cheng Kung University, Tainan, Taiwan; ^3^ Institute of Basic Medical Sciences, College of Medicine, National Cheng Kung University, Tainan, Taiwan; ^4^ Department of Internal Medicine, College of Medicine and Hospital, National Cheng Kung University, Tainan, Taiwan; ^5^ Department of Biochemistry and Molecular Biology, College of Medicine, National Cheng Kung University, Tainan, Taiwan; ^6^ Natural Products Research Laboratories, UNC Eshelman School of Pharmacy, University of North Carolina, Chapel Hill, NC, USA; ^7^ Laboratory of Pharmacognosy, Graduate School of Pharmaceutical Sciences, The University of Tokushima, Tokushima, Japan; ^8^ Chinese Medicine Research and Development Center, China Medical University and Hospital, Taichung, Taiwan; ^9^ Department of Pharmacology, College of Medicine, National Cheng Kung University, Tainan, Taiwan; ^10^ Graduate Institute of Medical Sciences, College of Medicine, Taipei Medical University, Taipei, Taiwan

**This article has been corrected:** The control data used in the assembly of Figure 5 was incorrect. After reviewing all of the raw data, an updated Figure 5 was created and is shown below. The authors declare that these corrections do not change the results or conclusions of this paper.

Original article: Oncotarget. 2015; 6:13671–13687. 13671-13687. https://doi.org/10.18632/oncotarget.3701

**Figure 5:**
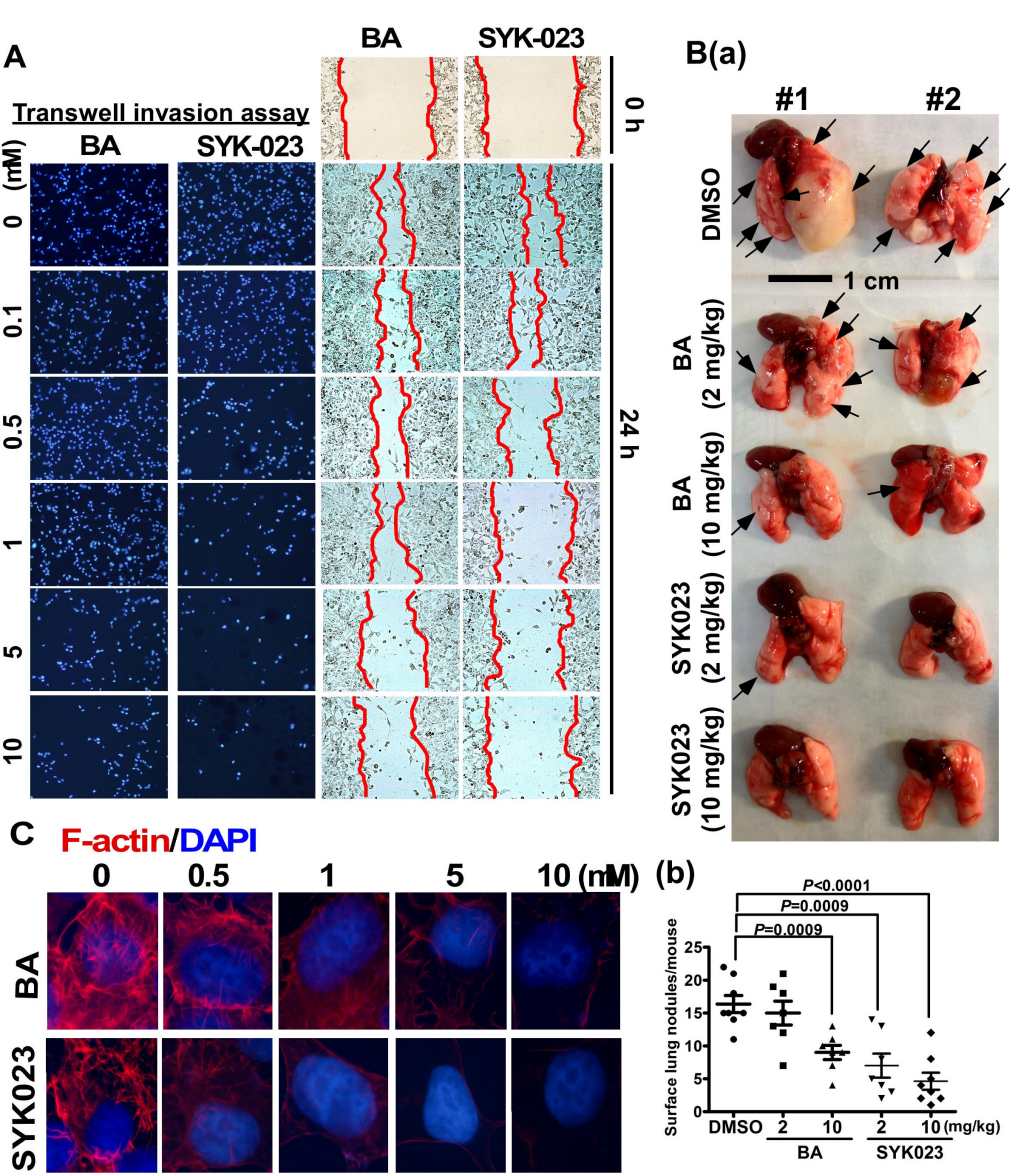
Effects of BA and SYK023 on lung tumor metastasis *in vitro *and *in vivo*. (**A**) After BA and SYK023 treatment for 36 h, H1299 cells were subjected to transwell invasion and wound-healing assay. (**B**) Representative images of lungs from SCID mice with metastasis (a). The number of surface lung tumors. Data are expressed as mean ± s.e.m, *P*-value is indicated (b). (**C**) H1299 cells on the coverslip were treated with the indicated drug, and subjected to immunofluorescent staining for F-actin and DAPI (1000×).

