# Supplementary material for: A novel derivative of betulinic acid, SYK023, suppresses lung cancer growth and malignancy
Source: Oncotarget. 2015 Mar 30;6(15):13671–87. doi: 10.18632/oncotarget.3701 (PMC4537041; doi:10.18632/oncotarget.3701)
Supplement: Supplementary file 1 [file oncotarget-06-13671-s001.pdf]

A novel derivative of betulinic acid, SYK023, suppresses lung cancer growth and malignancy

Supplementary Material

Supplementary Materials and Methods

Antibodies

Active caspase 3 (Cell Signaling Technology, Boston, MA, USA), active caspase 8 (Novus Biologicals, Littleton, CO, USA), active caspase 9 (Novus Biologicals), active caspase 12 (Millipore, Bedford, MA, USA), p-eIF2α (Cell Signaling Technology), cytochorm c (Santa Cruz Biotechnology, Dallas, Texas, USA), COX4 (Novus, Biologicals), cyclin A2 (Proteintech Group, Chicago, IL, USA), cyclin E2 (Santa Cruz Biotechnology), cyclin B1 (Santa Cruz Biotechnology), Ki-67 (Santa Cruz Biotechnology), Sp1 (Millipore), $p15^{ink4b}$ (Santa Cruz Biotechnology), $p21^{CIP1}$ (Millipore), p-FAK (abcam, Cambridge, MA, USA), FAK (abcam), p-Src (abcam), Src (abcam), p-Akt (abcam), Akt (abcam), p-mTOR (Cell Signaling Technology), PTEN (abcam), N-cadherin (Santa Cruz Biotechnology), β-catenin (Cell Signaling Technology), vimentin (abcam), c-myc (abcam), SYPD (Bioss Inc., Woburn, MA, USA)

Transfection

For SYPD knockdown, H1299 cells were transfected with SYPD shRNA using lipofectamine 2000 (Invitrogen) according to the manufacture's instruction. Fifty percent confluent cells were incubated with Opti-MEM (Invitrogen) containing 4 μg of shRNA, which was purchased from National RNAi Core Facility Platform (Taipei, Taiwan), for 8 h. After replacing the medium, cells were incubated with fresh medium for additional 24 h. Subsequently, cells were treated with the indicated drug or subjected to immunofluorescence.

Proliferation assay

After treatment with BA or derivatives for indicated times, cells were photographed and counted by hemacytometer.

Flow cytometry

For cell cycle analysis, ethanol-fixed cells were stained with 50 mg/ml propidium iodide (PI; QIAGEN, Valencia, CA, USA) for 30 min at 37℃, and analyzed by a flow cytometer (FACSCalibur; BD BioSciences, San Jose, CA, USA). For apoptosis analysis, annexin V assay kit (Invitrogen) was used. Trypsinized cells were stained with PI (100 mg/ml) and annexin V for 15 min at room temperature. Stained cells were analyzed by the Beckman Coulter Cell Lab Quanta SC Flow Cytometer (Beckman, Miami, FL, USA).

Microarray analysis

The RNA from H1299 cells was extracted by the Trizol reagent, and cDNA was subjected to microarray analysis by Phalanx Biotech (Hsinchu, Taiwan). The functional groups of microarray results were analyzed by the Gene Set Enrichment Analysis (GSEA) website.

3-*O*-(2-Chlorobezoyl)betulinic acid (SYK010)

2-Chlorobenzoic acid (275.5 mg, 1.77 mmol) was dissolved in CH_2Cl_2 (5.2 mL) and Et_3N (0.25 mL) with EDCI (274.2 mg, 1.77 mmol) and DMAP (108.6 mg, 0.89 mmol) for 20 min, before betulinic acid (75.6 mg, 0.17 mmol) was added in this solution. The reaction mixture was stirred for 1.5 h. Water was added to quench the reaction and CH_2Cl_2 was used for extraction and the organic layer was washed by brine, and dried over $MgSO_4$. After elimination the solvent, the residue was purified by Medium-pressure column chromatography with CH_2Cl_2/MeOH to give **SYK010** (45.0 mg, 46%) as white powder; ^{1}H NMR (400 MHz, $CDCl_3$): δ 7.80 (1H, dd, *J* = 1.5, 7.8 Hz, Ar-H-6), 7.44 (1H, dd, *J* = 1.5, 7.8 Hz, Ar-H-2), 7.40 (1H, dt, *J* = 1.5, 7.8 Hz, Ar-H-3), 7.30 (1H, dt, *J* = 1.5, 7.8 Hz, Ar-H-5), 4.76 (1H, dd, *J* = 4.9, 11.7 Hz, H-3), 4.75 and 4.62 (1H each, br s, H-29), 3.01 (1H, dt, *J* = 4.4, 10.7 Hz, H-19), 1.71 (3H, s, H-30), 1.00, 0.96, 0.57, 0.94, and 0.89 (3H each, s, 5 × CH_3); MS *m/z*: 593.45 $(M – H)^-$.

3-*O*-(1-Naphthoyl)betulinic acid (SYK012)

A solution of 1-naphthoic acid (300.3 mg, 1.75 mmol) in CH_2Cl_2 (5.0 mL) and Et_3N (0.25 mL) was treated with EDCI (274.9 mg, 1.77 mmol) and DMAP (107.7 mg, 0.88 mmol). Betulinic acid (74.7 mg, 0.16 mmol) was added in this solution, which was stirred for 1.5 h. Water was added and the mixture was extracted with CH_2Cl_2. After evaporation, the residue was eluted through Medium-pressure column chromatography with CH_2Cl_2/MeOH to give **SYK012** as white powder; ^{1}H NMR (400 MHz, $CDCl_3$): δ 8.95 (1H, br d, *J* = 8.8 Hz, Np-H-8), 8.16 (1H, dd, *J* = 1.0, 7.3 Hz, Np-H-2), 8.00 (1H, br d, *J* = 7.8 Hz, Np-H-4), 7.87 (1H, br d, *J* = 8.3 Hz, Np-H-5), 7.60 (1H, ddd, *J* = 1.5, 6.8, 8.8 Hz, Np-H-7), 7.55-7.45 (2H, m, Np-H-3, 6), 4.85 (1H, dd, *J* = 4.6, 11.5 Hz, H-3), 4.76 (1H, d, *J* = 1.4 Hz, H-29), 4.63 (1H, br s, H-29), 3.03 (1H, dt, *J* = 4.2, 10.6 Hz, H-19), 1.72 (3H, s, H-30), 1.01, 1.00, 1.00, 0.97, and 0.92 (3H each, s, 5 × CH_3); MS *m/z*: 609.70 $(M – H)^-$.

3-*O*-(4-Methoxybezoyl)betulinic acid (SYK016)

Betulinic acid (72.9 mg, 0.16 mmol) was treated with *p*-anisoyl chloride (60 μL, 0.44 mmol) in CH_2Cl_2 (2.8 mL) and Et_3N (0.5 mL) for 1 h. Then water was added to quench the reaction. The mixture was extracted with CH_2Cl_2, washed with brine, and dried over $MgSO_4$. The solvent was removed in vacuo, and the residue was chromatographed using CH_2Cl_2/MeOH to give **SYK016** as white powder; ^{1}H NMR (400 MHz, $CDCl_3$): δ 7.98 (2H, d, *J* = 8.8 Hz, Ar-H-2, 6), 6.96 (2H, d, *J* = 8.8 Hz, Ar-H-3, 5), 4.76 (1H, d, *J* = 2.0 Hz, H-29), 4.63 (1H, br s, H-29), 3.89 (3H, s, OCH_3), 3.19 (1H, dd, *J* = 4.9, 11.2 Hz, H-3), 3.05 (1H, dt, *J* = 4.9, 10.8 Hz, H-19), 1.71 (3H, s, H-30), 1.00, 0.99, 0.97, 0.83, and 0.76 (3H each, s, 5 × CH_3); MS *m/z*: 613.30 $(M + Na)^+$.

3-*O*-(3-Methoxybezoyl)betulinic acid (SYK017)

Betulinic acid (71.9 mg, 0.16 mmol) was dissolved with *m*-anisoyl chloride (60 μL, 0.43 mmol) in CH_2Cl_2 (3.0 mL) and Et_3N (0.5 mL). The reaction mixture was stirred for 1 h before water was added. The mixture was extracted with CH_2Cl_2, and the organic layers were washed with brine, and dried over $MgSO_4$. After elimination the solvent, the residue was purified by Medium-pressure column chromatography with CH_2Cl_2/MeOH to give **SYK017** as white powder ; ^{1}H NMR (400 MHz, $CDCl_3$): δ 7.61 (1H, br d, *J* = 7.8 Hz, Ar-H-6), 7.54 (1H, dd, *J* = 1.4, 2.7 Hz, Ar-H-2), 7.39 (1H, dd, *J* = 7.8, 8.3 Hz, Ar-H-5), 7.18 (1H, br dd, *J* = 2.7, 8.3 Hz, Ar-H-4), 4.76 and 4.63 (1H each, br s, H-29), 3.86 (3H, s, OCH_3), 3.19 (1H, dd, *J* = 5.1, 11.0 Hz, H-3), 3.04 (1H, dt, *J* = 4.9, 11.0 Hz, H-19), 1.71 (3H, s, H-30), 1.00, 0.99, 0.97, 0.83, and 0.76 (3H each, s, 5 × CH_3); MS *m/z*: 589.25 $(M – H)^-$.

3-*O*-(1,4-Benzodiozane-6-carboxyl)betulinic acid (SYK018)

A solution of 1,4-benzodioxane-6-carboxylic acid (194.1 mg, 1.08 mmol) in CH_2Cl_2 (3.8 mL) and Et_3N (0.5 mL) was treated with EDCI (169.5 mg, 1.09 mmol) and DMAP (63.2 mg, 0.52 mmol), and 20 min later betulinic acid (78.2 mg, 0.17 mmol) was added. The reaction mixture was stirred at room temperature for 1.5 h. Water was added to quench the reaction and the mixture was extracted with CH_2Cl_2, washed with brine, and dried over $MgSO_4$. The solvent was removed, and the crude was eluted through Medium-pressure column chromatography with *n*-hexane/EtOAc to give **SYK018** (17.4 mg, 16%) as white powder; ^{1}H NMR (400 MHz, $CDCl_3$): δ 7.58-7.53 (2H, m, Ar-H-2, 6), 6.88 (1H, d, *J* = 8.8 Hz, Ar-H-5), 4.75 and 4.62 (1H each, br s, H-29), 4.67 (1H, dd, *J* = 4.9, 10.7 Hz, H-3), 4.29 (4H, m, $-OCH_2CH_2O-$), 3.01 (1H, dt, *J* = 4.4, 10.7 Hz, H-19), 1.70 (3H, s, H-30), 0.99, 0.97, 0.96, 0.90, and 0.89 (3H each,

s, 5 × CH_3); MS *m/z*: 617.50 $(M – H)^-$.

3-*O*-(3,4,5-Trimethoxyphenylacetyl)betulinic acid (SYK019)

3,4,5-Trimethoxyphenylacetic acid (159.1 mg, 0.70 mmol) was treated with EDCI (108.7 mg, 0.68 mmol) and DMAP (52.8 mg, 0.43 mmol) in CH_2Cl_2 (2.5 mL) and Et_3N (0.25 mL) for 20 min. Betulinic acid (81.4 mg, 0.18 mmol) was added in this solution, and the mixture was stirred for 1 h. Water was added to quench the reaction and CH_2Cl_2 was used for extraction. The organic layer was washed by brine, and dried over $MgSO_4$. The crude product obtained after concentration was purified by Medium-pressure column chromatography with *n*-hexane/EtOAc to give **SYK019** (8.3 mg, 7%) as white powder; ^1H NMR (400 MHz, $CDCl_3$): δ 6.51 (2H, s, Ar-H-2, 6), 4.73 (1H, d, *J* = 2.0 Hz, H-29), 4.61 (1H, br s, H-29), 4.47 (1H, dd, *J* = 5.4, 10.8 Hz, H-3), 3.84 (6H, s, 2 × OCH_3), 3.82 (3H, s, OCH_3), 3.53 (1H, s, $OCOCH_2Ph$), 2.99 (1H, dt, *J* = 4.4, 10.7 Hz, H-19), 1.69 (3H, s, H-30), 0.96, 0.93, 0.84, 0.80, and 0.76 (3H each, s, 5 × CH_3); MS *m/z*: 663.65 $(M – H)^-$.

3-*O*-[3-(3,4,5-Trimethoxyphenyl)propionyl]betulinic acid (SYK021)

3-(3,4,5-Trimethoxyphenyl)propionic acid (267.9 mg, 1.12 mmol) was treated with EDCI (181.6 mg, 1.17 mmol) and DMAP (119.4 mg, 0.98 mmol) in CH_2Cl_2 (5.0 mL) and Et_3N (0.5 mL) for 20 min. Betulinic acid (49.7 mg, 0.11 mmol) was added in this solution, which was stirred for 7 h. Then water was added, and the mixture was extracted with CH_2Cl_2, and washed by brine, and dried over $MgSO_4$. After evaporation of the solvent, the residue was eluted through Medium-pressure column chromatography with *n*-hexane/EtOAc to give **SYK021** (5.2 mg, 7%) as white powder; ^1H NMR (400 MHz, $CDCl_3$): δ 6.42 (2H, s, Ar-H-2, 6), 4.74 (1H, d, *J* = 2.0 Hz, H-29), 4.61 (1H, br s, H-29), 4.48 (1H, dd, *J* = 6.1, 10.0 Hz, H-3), 3.84 (6H, s, 2 ×

OCH_3), 3.81 (3H, s, OCH_3), 3.00 (1H, dt, *J* = 4.4, 10.8 Hz, H-19), 2.90 (2H, m, $OCOCH_2CH_2Ph$), 2.62 (2H, d, *J* = 6.6, 8.5 Hz $OCOCH_2CH_2Ph$), 1.69 (3H, s, H-30), 0.97, 0.93, 0.84, 0.80, and 0.77 (3H each, s, 5 × CH_3); MS *m/z*: 677.55 $(M – H)^-$.

3-*O*-(3,4-Methylenedioxyphenylacetyl)betulinic acid (SYK022)

3,4-Methylenedioxyphenylacetic acid (97.9 mg, 0.54 mmol) was dissolved in CH_2Cl_2 (4.7 mL) and Et_3N (0.5 mL) with EDCI (93.0 mg, 0.60 mmol) and DMAP (58.2 mg, 0.48 mmol) for 2 h. Betulinic acid (48.7 mg, 0.11 mmol) was added in this solution. The reaction mixture was stirred overnight before water was added. The mixture was extracted with CH_2Cl_2, and the organic layer was washed by brine, and dried over $MgSO_4$. After elimination the solvent, the residue was purified by Medium-pressure column chromatography with CH_2Cl_2/MeOH to give **SYK022** (7.2 mg, 11%) as white powder; ^{1}H NMR (400 MHz, $CDCl_3$): δ 6.78 (1H, d, *J* = 1.5 Hz, Ar-H-2), 6.74 (1H, d, *J* = 7.8 Hz, Ar-H-5), 6.72 (1H, dd, *J* = 1.5, 7.8 Hz, Ar-H-6), 5.93 (2H, s, $-OCH_2O-$), 4.73 (1H, d, *J* = 2.0 Hz, H-29), 4.61 (1H, br s, H-29), 4.46 (1H, dd, *J* = 6.3, 10.3 Hz, H-3), 3.50 (1H, s, $OCOCH_2Ph$), 2.99 (1H, dt, *J* = 4.9, 10.7 Hz, H-19), 1.69 (3H, s, H-30), 0.96, 0.92, 0.83, 0.78, and 0.76 (3H each, s, 5 × CH_3); MS *m/z*: 617.50 $(M – H)^-$.

3-*O*-(4-Benzyloxyphenylacetyl)betulinic acid (SYK024)

A solution of 4-benzyloxyphenylacetic acid (136.9 mg, 0.57 mmol) in CH_2Cl_2 (4.8 mL) and Et_3N (0.5 mL) was treated with EDCI (87.8 mg, 0.56 mmol) and DMAP (62.3 mg, 0.51 mmol), and 20 min later betulinic acid (47.0 mg, 0.10 mmol) was added. The mixture was stirred overnight before water was added. Then CH_2Cl_2 was used for extraction, and the organic layers were washed with brine, and dried over $MgSO_4$. The solvent was evaporated, and the residue was eluted through

Medium-pressure column chromatography with *n*-hexane/EtOAc to give **SYK024** (9.2 mg, 13%) as colorless oil; ^{1}H NMR (400 MHz, $CDCl_3$): δ 7.45-7.24 (5H, m, benzyl Ar-H-2—5), 7.19 (2H, d, *J* = 8.8 Hz, phenylacetyl Ar-H-2, 6), 6.92 (2H, d, *J* = 8.8 Hz, phenylacetyl Ar-H-3, 5), 5.05 (2H, s, benzyl CH_2), 4.73 and 4.60 (1H each, br s, H-29), 4.45 (1H, dd, *J* = 5.9, 10.2 Hz, H-3), 2.99 (1H, m, H-19), 1.69 (3H, s, H-30), 0.96, 0.92, 0.83, 0.77, and 0.73 (3H each, s, 5 × CH_3); MS *m/z*: 679.65 $(M – H)^-$.

3-*O*-(2-Methoxycinnamoyl)betulinic acid (SYK025)

2-Methoxycinnamic acid (121.5 mg, 0.68 mmol) was treated with EDCI (110.0 mg, 0.71 mmol) and DMAP (64.6 mg, 0.53 mmol) in CH_2Cl_2 (3.7 mL) and Et_3N (1.0 mL) for 40 min. Then betulinic acid (44.5 mg, 0.10 mmol) was added in this solution. The mixture was stirred overnight before water was added. The crude was extracted with CH_2Cl_2, and the organic layer was washed by brine, and dried over $MgSO_4$. The solvent was removed, the residue was purified by Medium-pressure column chromatography with *n*-hexane/EtOAc to give **SYK025** (9.9 mg, 16%) as white powder; ^{1}H NMR (400 MHz, $CDCl_3$): δ 7.98 (1H, d, *J* = 16.4 Hz, cinnamoyl H-7), 7.51 (1H, dd, *J* = 1.5, 7.8 Hz, Ar-H-6), 7.34 (1H, dt, *J* = 1.5, 7.8 Hz, Ar-H-4), 6.95 (1H, br t, *J* = 7.8 Hz, Ar-H-5), 6.91 (1H, br d, *J* = 7.8 Hz, Ar-H-3), 6.51 (1H, d, *J* = 16.4 Hz, cinnamoyl H-8), 4.75 (1H, d, *J* = 1.5 Hz, H-29), 4.62 (1H, br s, H-29), 4.62 (1H, m, H-3), 3.88 (3H, s, OCH_3), 3.01 (1H, dt, *J* = 4.4, 10.8 Hz, H-19), 1.70 (3H, s, H-30), 0.99, 0.95, 0.92, 0.89, and 0.88 (3H each, s, 5 × CH_3); MS *m/z*: 615.50 $(M – H)^-$.

3-*O*-(4-Methoxycinnamoyl)betulinic acid (SYK026)

4-Methoxycinnamic acid (118.1 mg, 0.66 mmol) was dissolved in CH_2Cl_2 (4.2 mL) and Et_3N (1.0 mL) with EDCI (104.9 mg, 0.68 mmol) and DMAP (70.6 mg, 0.58

mmol) for 30 min. Then betulinic acid (44.3 mg, 0.10 mmol) was added in this solution, which was stirred overnight. Water was added to quench the reaction. The mixture was extracted with CH_2Cl_2, and washed by brine, and dried over $MgSO_4$. The solvent was removed by evaporation, the residue was eluted through Medium-pressure column chromatography with *n*-hexane/EtOAc to give **SYK026** as white powder; ^{1}H NMR (400 MHz, $CDCl_3$): δ 7.62 (1H, d, *J* = 15.6 Hz, cinnamoyl H-7), 7.48 (2H, d, *J* = 8.8 Hz, Ar-H-2, 6), 6.90 (2H, d, *J* = 8.8 Hz, Ar-H-3, 5), 6.31 (1H, d, *J* = 15.6 Hz, cinnamoyl H-8), 4.74 (1H, d, *J* = 1.4 Hz, H-29), 4.62 (1H, br s, H-29), 4.61 (1H, m, H-3), 3.84 (3H, s, OCH_3), 3.01 (1H, dt, *J* = 4.4, 10.8 Hz, H-19), 1.70 (3H, s, H-30), 0.99, 0.95, 0.91, 0.89, and 0.88 (3H each, s, 5 × CH_3); MS *m/z*: 615.70 $(M – H)^-$.

3-*O*-(3,4-Dimethoxycinnamoyl)betulinic acid (SYK027)

3,4-Dimethoxycinnamic acid (162.5 mg, 0.78 mmol) was dissolved in CH_2Cl_2 (5.0 mL) and Et_3N (1.0 mL) with EDCI (120.9 mg, 0.78 mmol) and DMAP (80.2 mg, 0.66 mmol) for 1 h. Betulinic acid (42.7 mg, 0.09 mmol) was added in this solution, which was stirred overnight. Water was added to quench the reaction, and then CH_2Cl_2 was used for extraction. The mixture was washed by brine, and dried over $MgSO_4$. The reaction product was purified by Medium-pressure column chromatography with *n*-hexane/EtOAc to give **SYK027** (7.3 mg, 13%) as white powder; ^{1}H NMR (400 MHz, $CDCl_3$): δ 7.60 (1H, d, *J* = 15.9 Hz, cinnamoyl H-7), 7.10 (1H, dd, *J* = 2.0, 8.3 Hz, Ar-H-6), 7.06 (1H, d, *J* = 2.0 Hz, Ar-H-2), 6.86 (1H, d, *J* = 8.3 Hz, Ar-H-5), 6.31 (1H, d, *J* = 15.9 Hz, cinnamoyl H-8), 4.75 (1H, d, *J* = 2.0 Hz, H-29), 4.62 (1H, br s, H-29), 4.62 (1H, m, H-3), 3.92 and 3.91 (3H each, s, 2 × OCH_3), 3.01 (1H, dt, *J* = 4.7, 10.6 Hz, H-19), 1.70 (3H, s, H-30), 0.99, 0.95, 0.93, 0.90, and 0.89 (3H each, s, 5 × CH_3); MS *m/z*: 645.60 $(M – H)^-$.

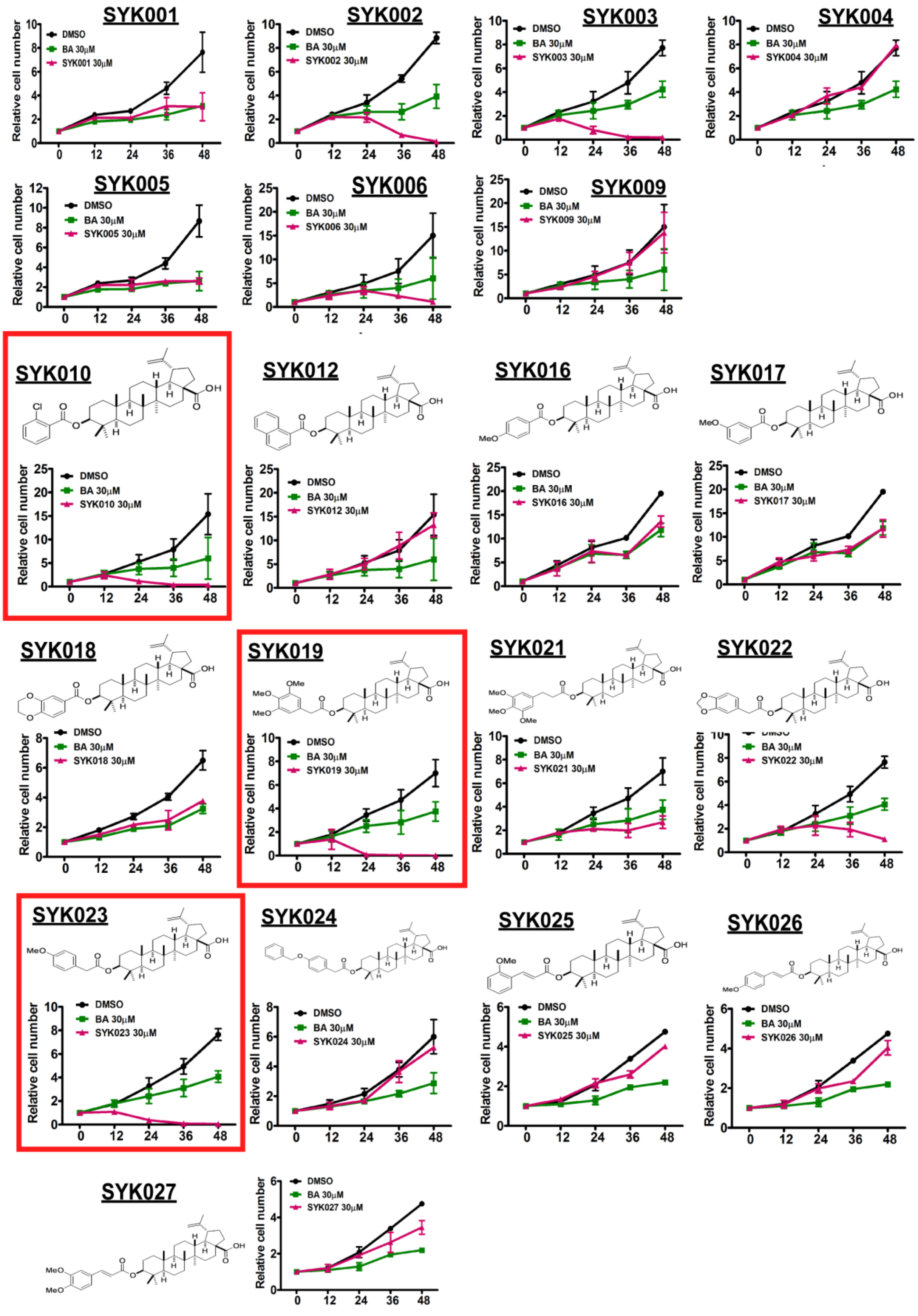

Supplementary Figure S1. Effects of BA and its derivatives on cell proliferation. After treatment with the indicated drug at 30 μM for the indicated period, cells were counted by hemacytometer.

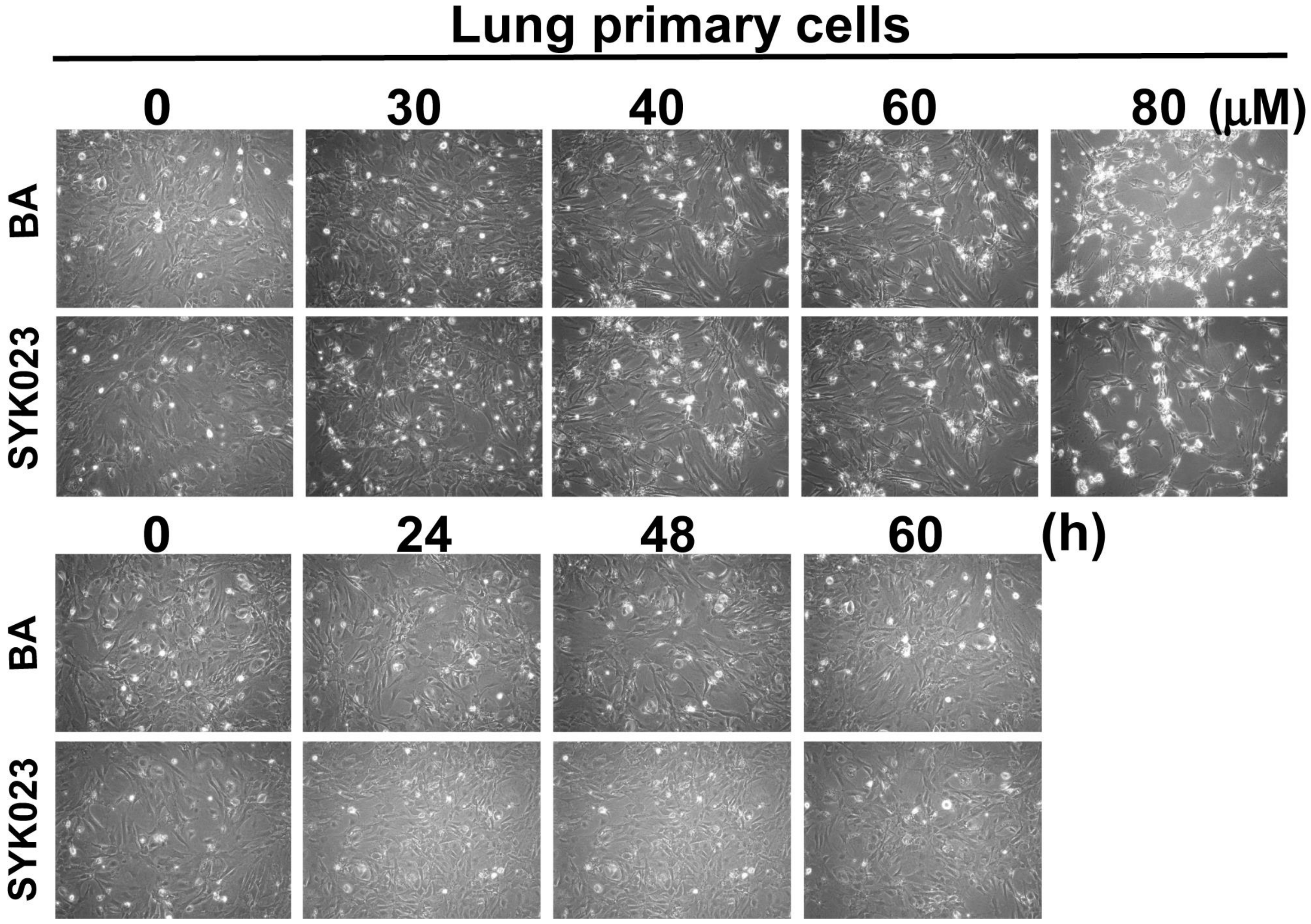

Supplementary Figure S2. Time- and dose-dependent effects of SYK023 on the survival of mouse lung primary cells. After treatment, cells were photographed by microscope under 100x magnification.

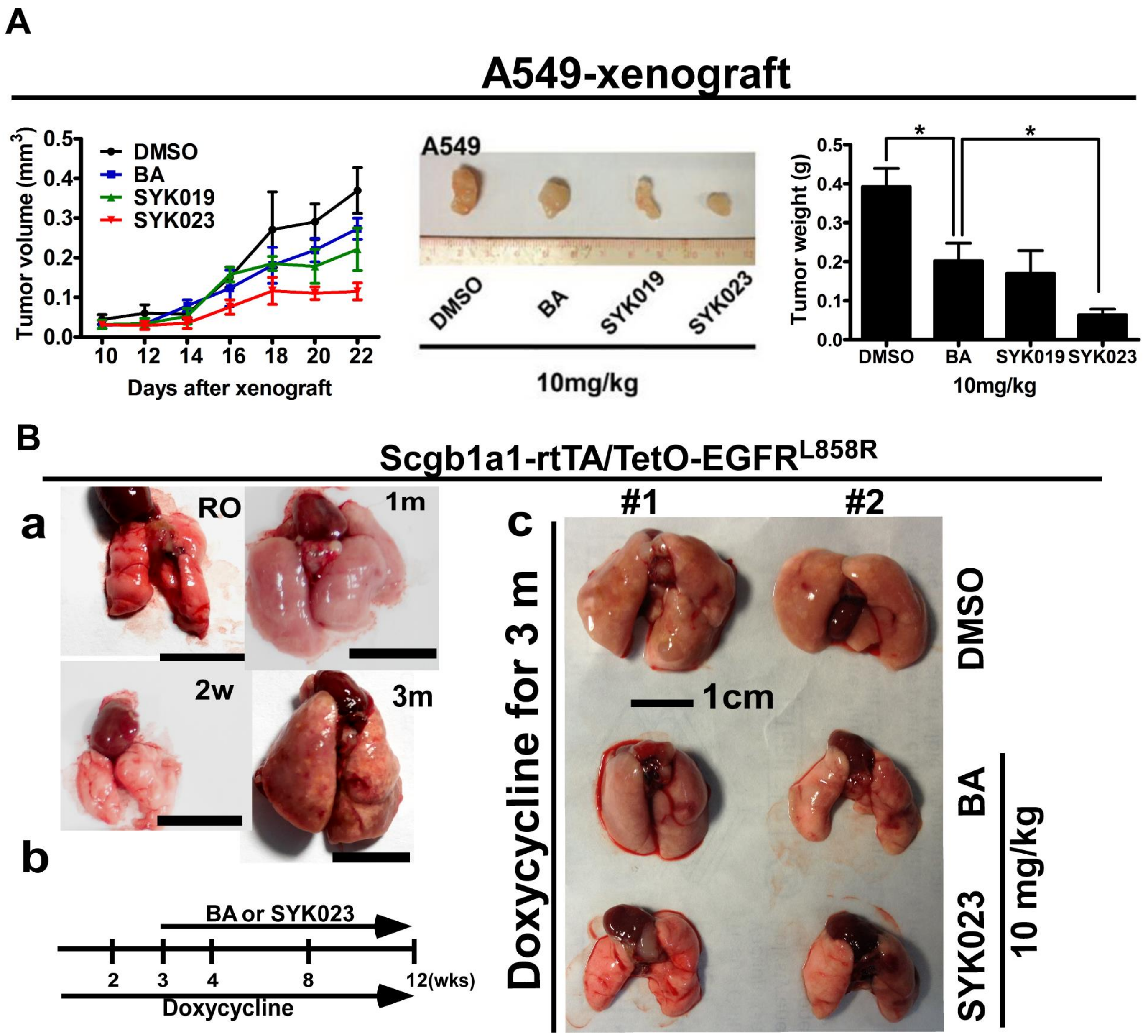

Supplementary Figure S3. Effect of SYK023 on tumor growth *in vivo*. A. A549-mediated xenograft model. Left, the growth of tumor was measured once two days; center, the images of excised tumors; right, each excised tumor was weighted. B. Lung tumors developed spontaneously under the control of doxycycline. a. The image of excised lung from EGFR^{L858R} mice treated with doxycycline for the indicated time. b. The time period of drug administration. c. The image of lungs from DMSO-, BA- or SYK023-treated EGFR^{L858R} mice.

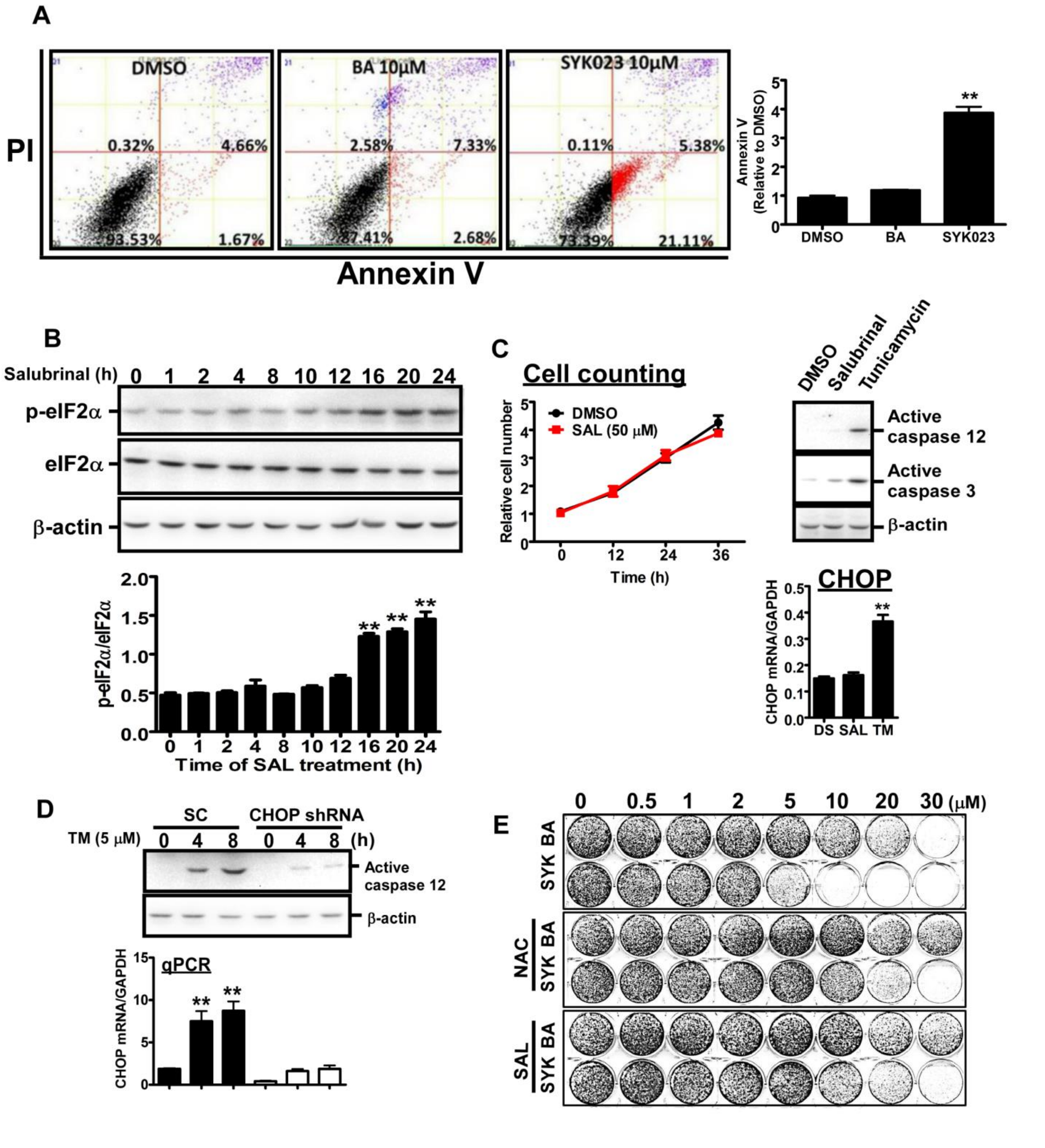

Supplementary Figure S4. A. The evaluation of SYK023-induced apoptosis by annexin V staining followed by flow cytometry. B. Effect of SAL on eIF2α phosphorylation. C. Effect of SAL on cell survival and apoptosis. D. SAL prevented tunicamycin (TM)-induced ER stress. E. Colony formation assay.

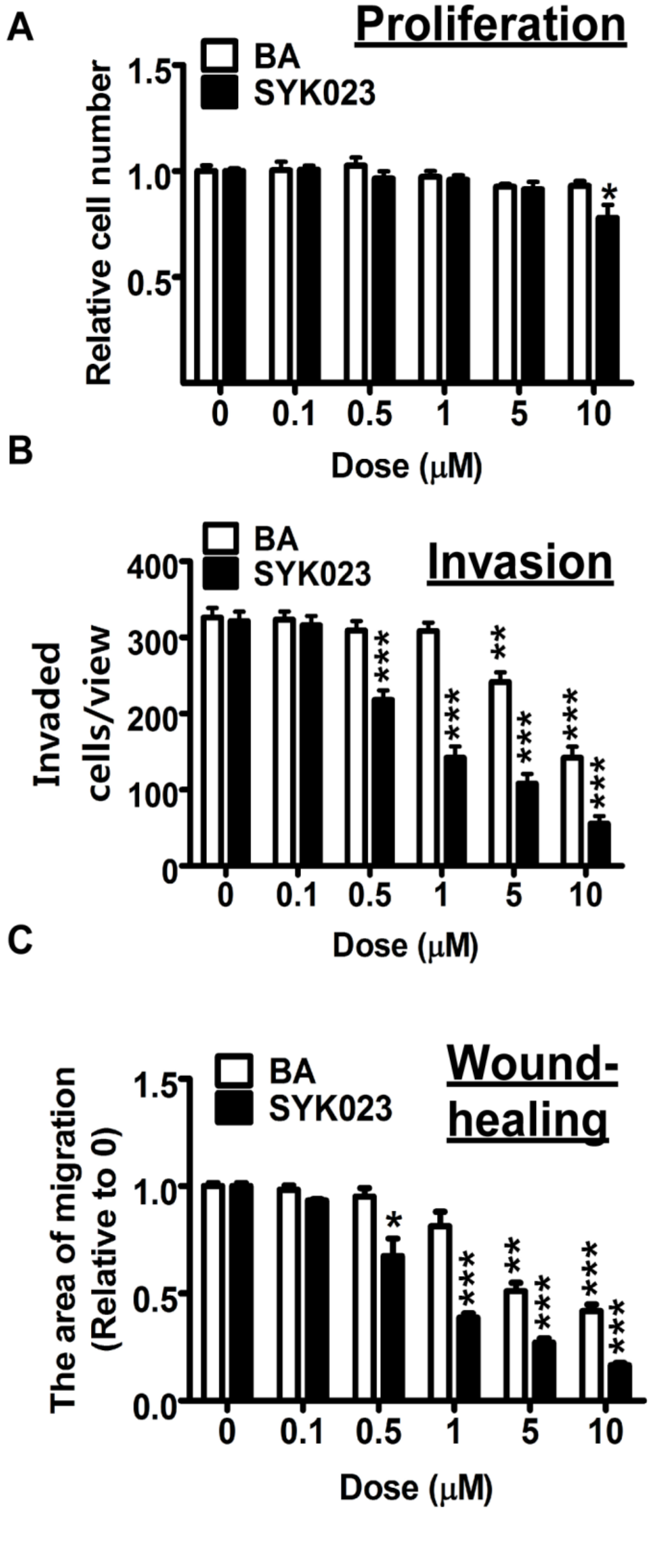

Supplementary Figure S5. Effect of SYK023 on tumor properties in H1299 cells. A. After treatment for 36 h, cells were counted by hemacytometer. B. BA- and SYK023-treated cells (2 x 10^4) were seeded onto the transwell plate for transwell migration. C. The quantative result for migratory area of cells. Data are expressed as mean±s.e.m. *P<0.05, **P<0.01, ***P<0.001.

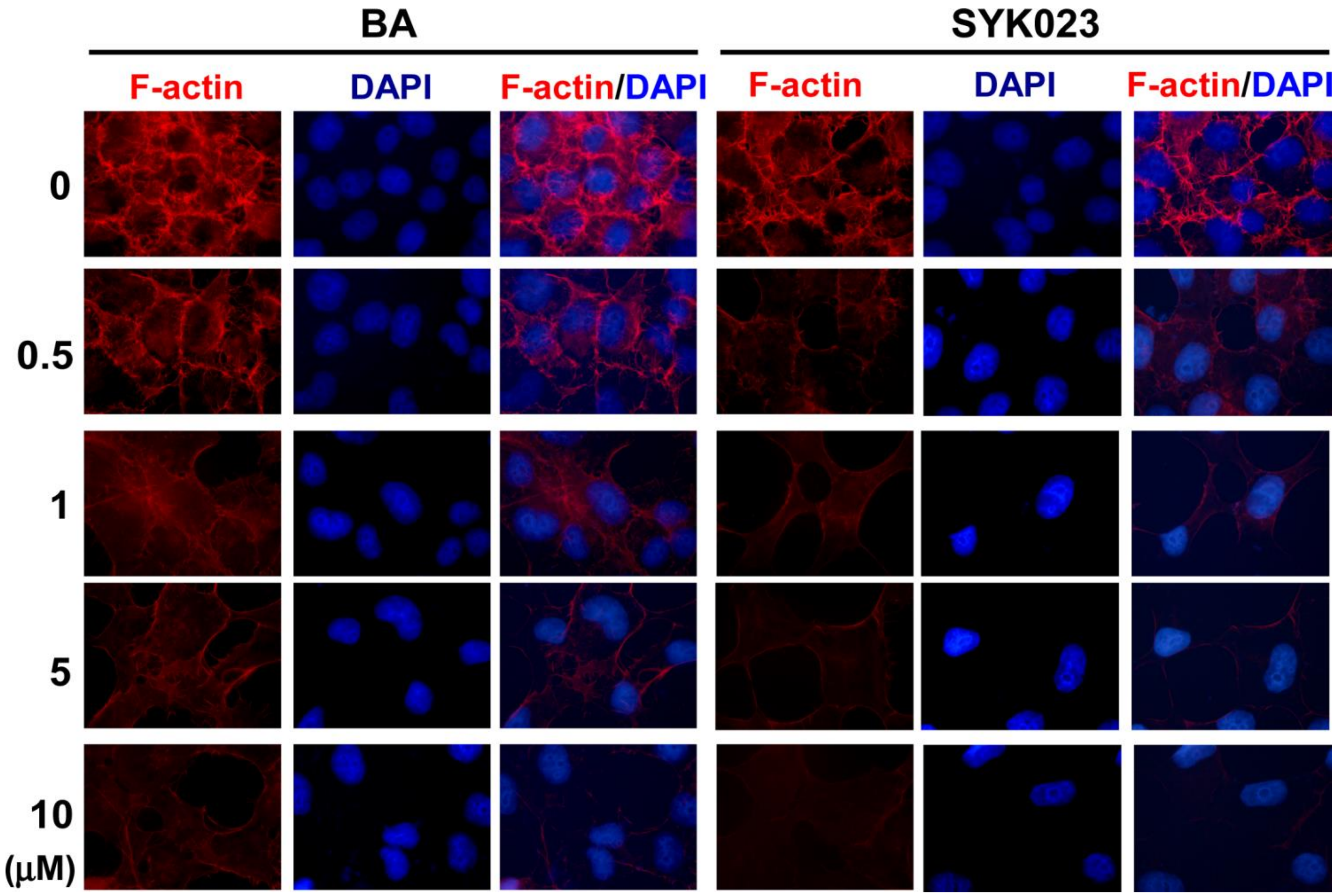

Supplementary Figure S6. Effect of SYK023 on F-actin polymerization. After treatment with the indicated dose of drug, fixed H1299 cells were immunostained by Alexa Fluor® 568 phalloidin and DAPI.

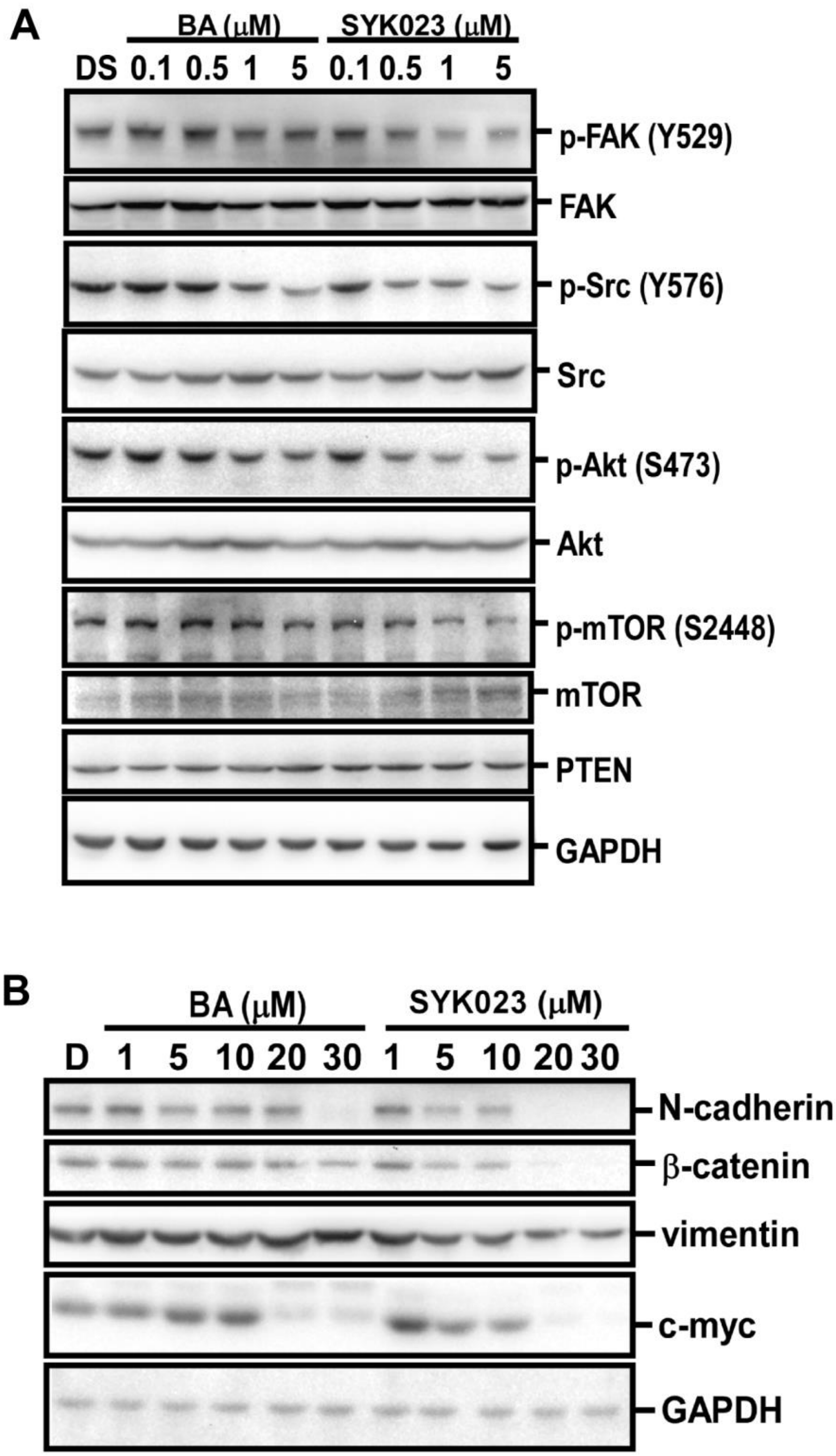

Supplementary Figure S7. Effect of SYK023 on expressions of proteins related to tumor malignancy.

A. Effect of SYK023 on F-actin stabilization-related proteins. After treatment for 36 h, H1299 cells were harvested for Western blotting using the antibody against p-FAK (Y529), FAK, p-Src (Y576), Src, p-Akt (S473), Akt, p-mTOR (S2448), mTOR, PTEN or GAPDH. B. Proteins related to EMT were also evaluated, including N-cadherin, β-catenin, vimentin or c-myc.

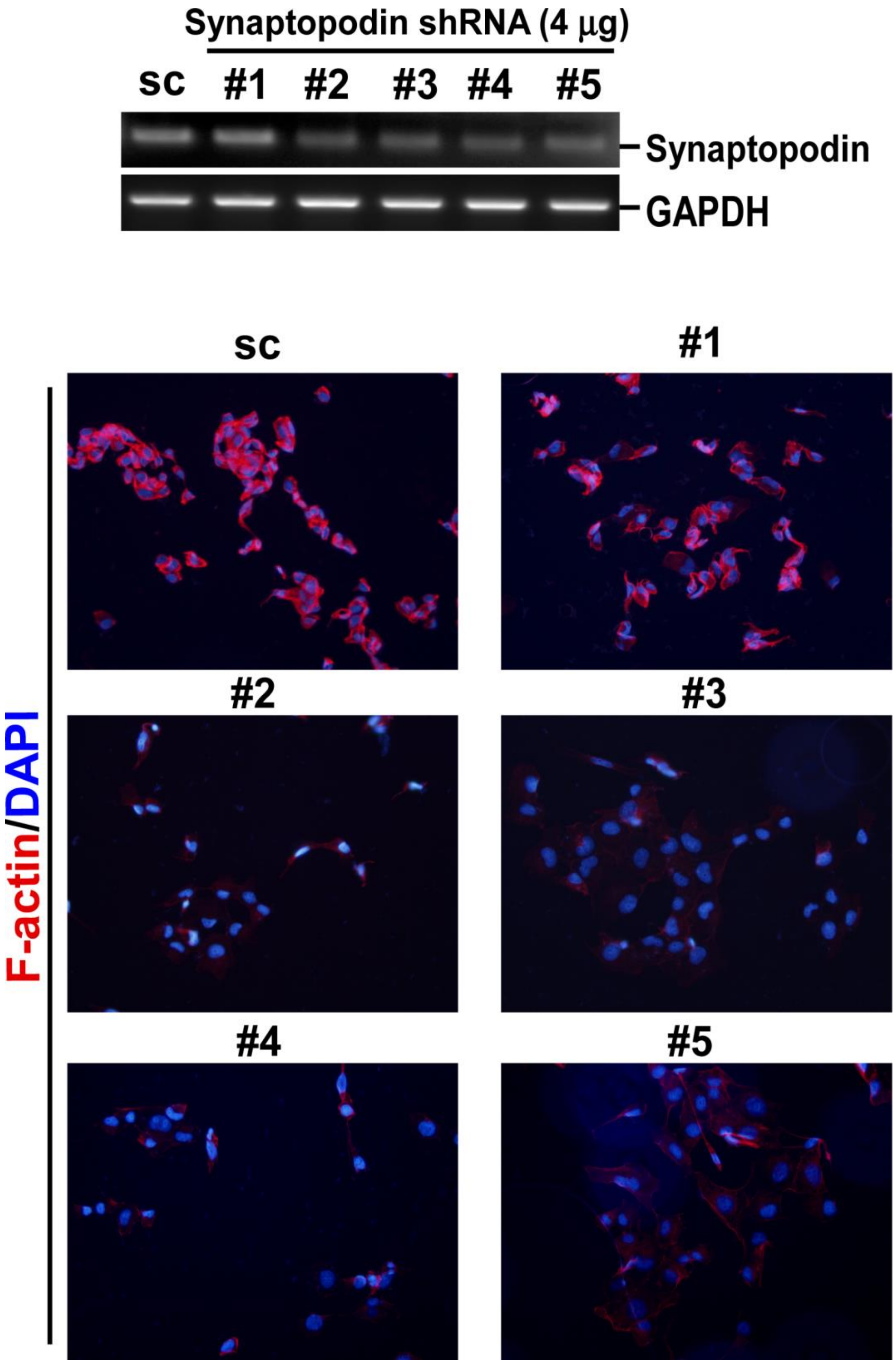

Supplementary Figure S8. Effect of SYPD knockdown on F-actin polymerization. A. The mRNA level of SYPD with or without shRNA-mediated knockdown. B. After transfection with scramble or SYPD shRNA for 48 h, H1299 cells were stained by Alexa Fluor® 568 phalloidin and DAPI.

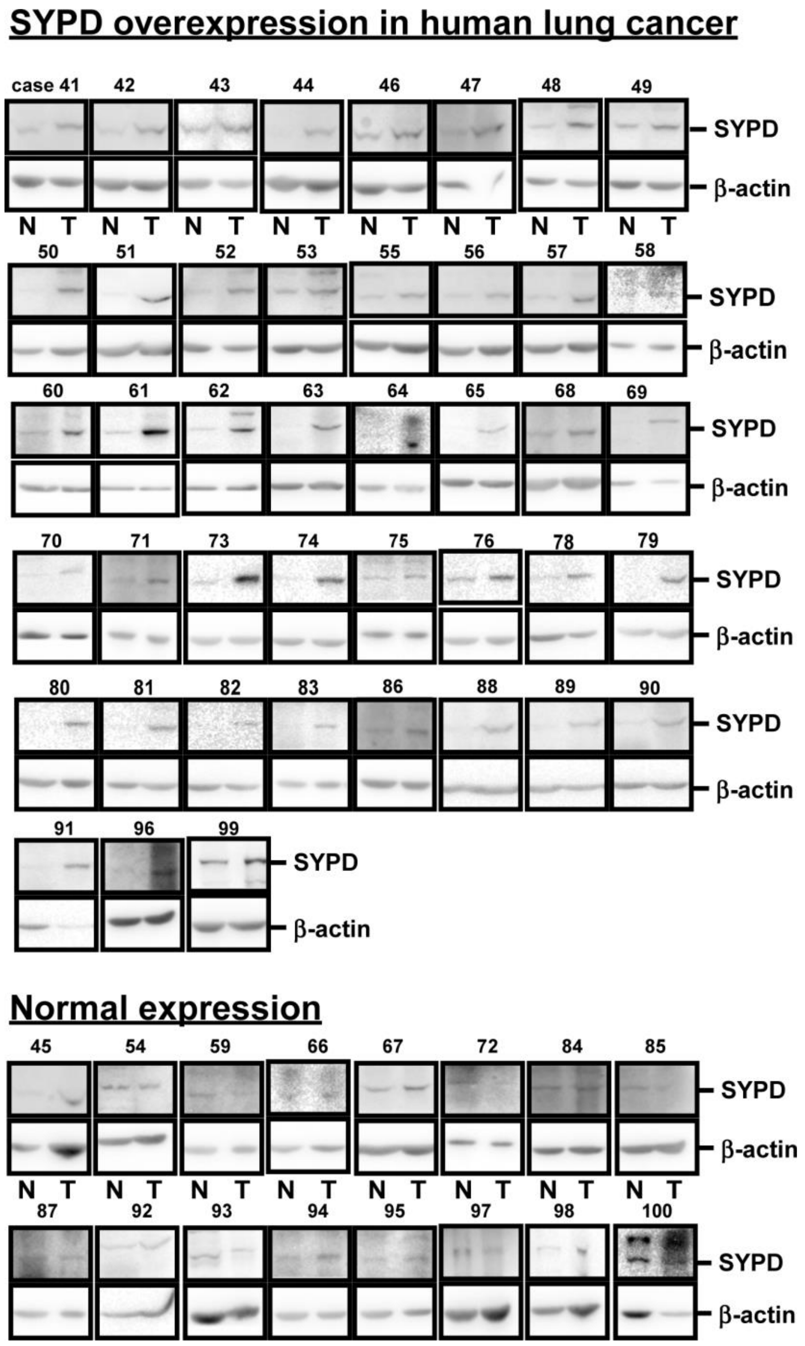

Supplementary Figure S9. The protein expression of SYPD in normal lung tissue and lung tumors from patients with lung cancer. Fifty-nine paired specimens were homogenized as protein lysates for Western blotting using the anti-SYPD antibody. Compared with normal tissue, higher expression of SYPD in lung tumor was defined as overexpression.

Supplementary Table S1. The number of survival and dead mice receiving drug administration.

Kras^{G12D} transgenic mice			
**Injection for 2 m (once/ 3 days)**	**Death during incubation period**	**Live**	**Total**
DMSO (100 µl)	0	8	8
BA (20 mg/kg)	4	8	12
SYK023 (20 mg/kg)	0	8	8
BA (10 mg/kg)	0	8	8
SYK023 (10 mg/kg)	0	8	8

SCID mice			
**Injection for 2 m (once/3 days)**	**Death during incubation period**	**Live**	**Total**
DMSO (100 µl)	0	12	12
BA (20 mg/kg)	5	7	12
SYK023 (20 mg/kg)	2	10	12
SYK019 (20 mg/kg)	6	6	12
BA (10 mg/kg)	0	6	6
SYK023 (10 mg/kg)	0	6	6
SYK019 (10 mg/kg)	2	4	6

Supplementary Table S2. Primers used for Q-PCR

CDC25a	F: GTCTCCACCTGGAAGTACAA<br>R: CCAGGGATAAAGACTGATGA
CDC25b	F: AAAGGATGATGATGCAGTTC<br>R: CTGTACATGACGAGGTCCTT
CCND3	F: CATCGAAAAACTGTGCATC<br>R: AATCATGTGCAATCACAGC
$p16^{INK4a}$	F: AGGGTTTTCTTGGTGAAGTT<br>R: CCCTCTTCTCAAGATCCTCT
CDC6	F: AACTAGCCAAAGTTCACCAA<br>R: GCAGATTCTTTCTTCAGTGG
$p57^{KIP2}$	F: ACGATGGAGCGTCTTGTC<br>R: GTAATCCCAGCGGTTCTG
PCNA	F: AACCAGGAGAAAGTTTCAGA<br>R: ATATACGTGCAAATTCACCA
SYPD	F: ACACCAACTTCTAACAGCAG<br>R: TTCTGTTGGATGCTAGAAAG
CHOP	F: TCACTCTCCAGATTCCAGTC<br>R: CTGTTCTTTCTCCTTCATGC
